# Development of *Safirinium* dyes for new applications: fluorescent staining of bacteria, human kidney cells, and the horny layer of the *epidermis*

**DOI:** 10.1038/s41598-022-19262-w

**Published:** 2022-09-05

**Authors:** Joanna Fedorowicz, Dagmara Bazar, Wioletta Brankiewicz, Hanna Kapica, Krzesimir Ciura, Beata Zalewska-Piątek, Rafał Piątek, Krzysztof Cal, Krystyna Mojsiewicz-Pieńkowska, Jarosław Sączewski

**Affiliations:** 1 Drug Research Program, Division of Pharmaceutical Biosciences, Faculty of Pharmacy, University of Helsinki, P.O. Box 56 (Viikinkaari 5 E), 00014 Helsinki, Finland; 2 Department of Chemical Technology of Drugs, Faculty of Pharmacy, Medical University of Gdańsk, Al. Gen. J. Hallera 107, 80-416 Gdańsk, Poland; 3 Department of Physical Chemistry, Medical University of Gdańsk, Al. Gen. Hallera 107, 80-416 Gdańsk, Poland; 4 Department of Pharmaceutical Technology and Biochemistry, Chemical Faculty, Gdańsk University of Technology, Narutowicza 11/12, 80-233 Gdańsk, Poland; 5 QSAR Lab Ltd., Trzy Lipy 3 St., 80-172 Gdańsk, Poland; 6 Department of Molecular Biotechnology and Microbiology, Chemical Faculty, Gdańsk University of Technology, Narutowicza 11/12, 80-233 Gdańsk, Poland; 7 BioTechMed Center, Gdańsk University of Technology, Narutowicza 11/12, 80-233 Gdańsk, Poland; 8 Department of Pharmaceutical Technology, Medical University of Gdańsk, Al. Gen. J. Hallera 107, 80-416 Gdańsk, Poland; 9 Department of Organic Chemistry, Faculty of Pharmacy, Medical University of Gdańsk, Al. Gen. J. Hallera 107, 80-416 Gdańsk, Poland

**Keywords:** Chemical tools, Medicinal chemistry, Imaging, Fluorescence imaging, Microscopy, Confocal microscopy, Phase-contrast microscopy

## Abstract

Low-molecular synthetic fluorophores are convenient tools in bioimaging applications. Several derivatives of *Safirinium* dyes as well as their reactive *N*-hydroxysuccinimide (NHS) esters bearing diverse substituents were synthesized and evaluated experimentally in terms of their lipophilicity by means of reverse-phase and immobilized artificial membrane high-performance liquid chromatography. Subsequently, the selected compounds were employed as novel cellular imaging agents for staining Gram-positive and Gram-negative bacteria, human kidney cell line, as well as human skin tissue. The analyzed dyes allowed for visualization of cellular structures such as mitochondria, endoplasmic reticulum, and cellular nuclei. They proved to be useful in fluorescent staining of *stratum corneum*, especially in the aspect of xenobiotic exposure and its penetration into the skin. The best results were obtained with the use of moderately lipophilic NHS esters of *Safirinium Q*. The development of *Safirinium* dyes is a promising alternative for commercially available dyes since the reported molecules have low molecular masses and exhibit efficient staining and remarkable water solubility. Moreover, they are relatively simple and low-cost in synthesis.

## Introduction

Fluorescent sensors and probes are of great interest due to their versatile applicability in chemical, environmental, and biological sciences^1–10^. Despite the widespread use of small organic fluorophores, the number of molecular scaffolds having tunable emission wavelengths and adjustable lipophilicity is limited^11,12^. The rational design of molecular probes is still a challenging task due the complexity of the processes underlying photophysical phenomena. Thus, the development of novel fluorescent molecules able to stain biological structures is an issue of major importance^10,13^.

Recently we have reported synthesis and optical properties of novel low molecular weight UV-fluorescent dyes bearing fused pyridino- or quinolino-triazolinium systems named *Safirinium P* or *Q*, respectively^14–16^. The developed dyes display high fluorescence quantum yields, large Stokes shifts, as well as distinct absorption and emission spectra. Moreover, these compounds demonstrate remarkable water solubility with no dependence of optical properties on pH, and lack of toxicity. The carboxyl group present within the structure of the dye can be easily converted into *N*-hydroxysuccinimide (NHS) ester form, which is reactive toward nucleophiles. This approach has been successfully applied for fluorescent staining of *Bacillus subtilis* spores as well as labeling of lysine-containing peptides and proteinogenic amino acids^14,17,18^. In addition, the conjugation of *Safirinium* core to fluoroquinolone antibiotic or triterpenoic acid resulted in the development of new hybrid dual-acting antibacterial agents with enhanced penetration into the bacterial cells^19–21^ or cytotoxic agents targeting endoplasmic reticulum^22^, respectively. *Safirinium* compounds were also used as quaternary ammonium ionization tags (ITs) that enable peptides identification as a result of the presence of a permanent positive charge localized on the nitrogen atom within the triazolinium moiety^17,18^.

The advantageous features of *Safirinium* derivatives prompted us to exploit their potential usage in staining of biological structures by means of fluorescence microscopy. Lipophilicity and (phospho)lipophilicity parameters for the novel and previously reported fluorescent *Safirnium*-type labeling tags were assessed experimentally with chromatographic methods and compared with these assessed for commercially available dyes, namely fluorescein and sulforhodamine B. The most promising compounds were tested for their applicability in visualization of bacteria, human kidney cells, and skin tissue.

## Results and discussion

The presented work is focused on the targeted modification of *Safirinium* chromophore chemical structure and selection of fluorescent probes that can efficiently stain biological structures.

### Chemistry

**Scheme 1:**
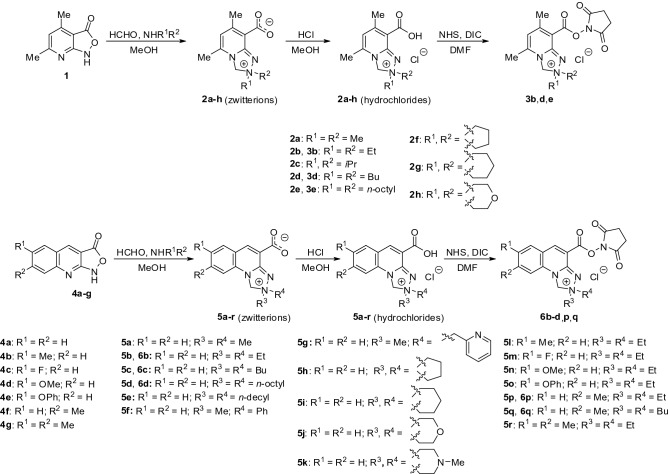
The synthesis of the *Safirinium* dyes.

A series of *Safirinium P* (**2a**–**h**) and *Q* (**5a**–**r**) dyes bearing diverse alkyl substituents within the triazolium and quinoline moieties were designed and synthesized to attain the structures with optimal staining properties (Scheme 1). 2,2-Dialkyl derivatives of 5,7-dimethyl-2,3-dihydro-[1,2,4]triazolo[4,3-*a*]pyridin-2-ium-8-carboxylate (**2a**–**h**) and 1,2-dihydro-[1,2,4]triazolo[4,3-*a*]quinolin-2-ium-4-carboxylate (**5a**–**r**) were obtained in the fluorogenic tandem Mannich—electrophilic amination reactions^14–16^ with use of two types of profluorophores, i.e. 4,6-dimethylisoxazolo[3,4-*b*]pyridin-3(1*H*)-one **1** and isoxazolo[3,4-*b*]quinolin-3(1*H*)-ones **4a**–**g**, respectively. Various secondary amines were utilized in order to modify lipophilicity of the final products. The obtained zwitterionic dyes were converted into hydrochlorides and subsequently reacted in anhydrous dimethylformamide (DMF) with *N*-hydroxysuccinimide (NHS) in the presence of *N*,*N*’-diisopropylcarbodiimide (DIC) as a coupling agent to yield a series of amine-reactive *Safirinium P* (**3b**,**d**,**e**) and *Q* (**6b**–**d**,**p**,**q**) esters. The structures of the products were confirmed with MS, IR, as well as NMR data (Supplementary Figs. S1–S11).

### Lipophilicity evaluation

**Figure 1:**
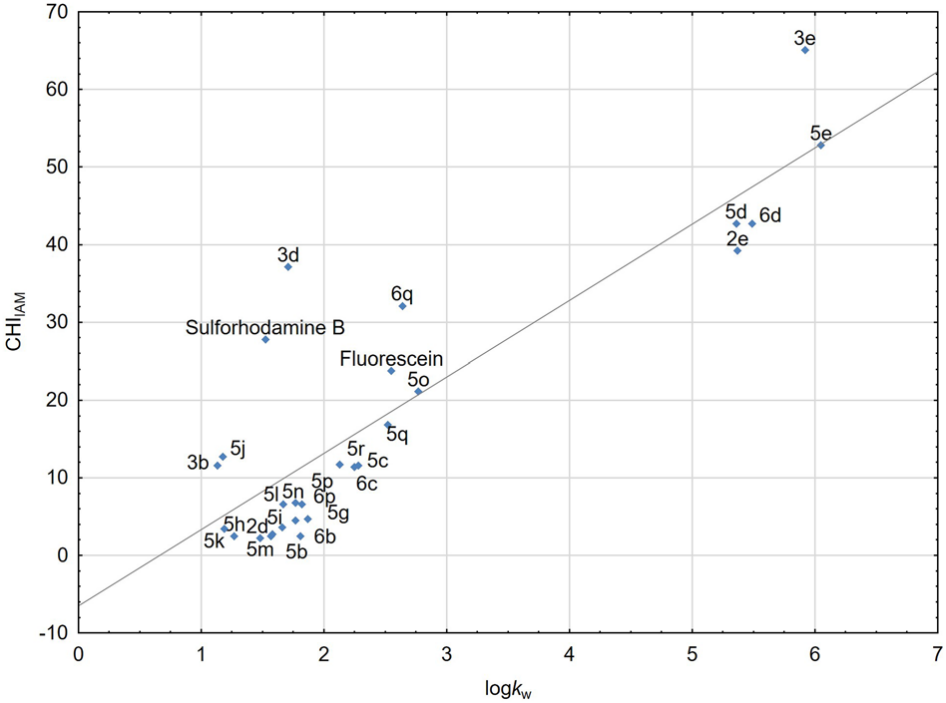
The scatterplot comparing CHI_IAM_ and log*k*_w_ indices of investigated fluorescent dyes.

Lipophilicity of a molecule should be considered when developing new dyes since it is a crucial determinant of the ability to penetrate the lipid bilayer of cellular membranes. Generally, lipophilicity can be assessed using theoretical and experimental approaches. Nevertheless, several studies indicated that calculation methods may lead to unsatisfactory results^20,23,24^. Consequently, experimental methods are still preferred. According to the Organization of Economic Co-operation and Development: shake-flask, reversed-phase high-performance liquid chromatography (RP-HPLC), and slow-stirring methods are recommended for lipophilicity determination^25^. Currently, among these methods, the reversed phase high-performance liquid chromatography (RP-HPLC) is currently the most popular tool for lipophilicity assessment^26^. RP-HPLC provides reproducible results in the course of relatively short analysis, combining full automation and reduction of organic solvents expenditure compared to the traditional shake-flask procedure^27^. Moreover, the RP-HPLC approach can be augmented with application of biosimilar stationary phases, such as immobilized artificial membrane (IAM), that are commercially available nowadays. The chromatographically determined lipophilicity and (phospho)lipophilicity indices for the investigated dyes are summarized in Table 1. Among the tested compounds, derivatives with long alkyl, *n*-octyl and *n*-decyl, substituents on the quaternary nitrogen atom (**2e**, **3e**, **5d**,**e** and **6d**) proved the highest values of (phospho)lipophilicity. Contrary, molecules **2a**–**c**, **f**–**h** and **5a**,**f** exhibit distinct hydrophilic character, as indicated by low log*k*_w_ values_._ Consequently, the latter compounds showed marginal affinity to phospholipids and migrated with the front of the mobile phase in IAM-HPLC. All these derivatives have a carboxyl group, which ionization can lead to the reduction of affinity to phospholipids. Generally, a molecule should have optimal lipophilicity values and affinity for phospholipids to effectively penetrate the biological membranes. CHI_IAM_ and log*k*_w_ indices of the tested substances and two reference dyes, fluorescein (log*k*_w_ = 2.55; CHI_IAM_ = 23.80) and sulforhodamine B (log*k*_w_ = 1.52; CHI_IAM_ = 27.80) are plotted in Fig. 1. The reference dyes demonstrate moderate lipophilicity and affinity to phospholipids. It has been also evidenced that several of the designed substances present similar physicochemical properties to the reference dyes and hence establish promising candidates for further evaluation. Close inspection of Fig. 1 reveals that lipophilicity and affinity to phospholipids within the tested group are intercorrelated with high correlation coefficient (r > 0.874).

**Table 1:** The chromatographically determined lipophilicity and (phospho)lipophilicity indices of the investigated and reference dyes.

Compound	log*k*_w_	CHI_IAM_
**2a**	1.11	ND
**2b**	1.13	ND
**2c**	1.22	ND
**2d**	1.57	2.50
**2e**	5.37	39.20
**2f**	1.14	ND
**2g**	1.19	ND
**2h**	1.03	ND
**3b**	1.13	11.60
**3d**	1.71	37.10
**3e**	5.92	65.10
**5a**	1.18	ND
**5b**	1.58	2.70
**5c**	2.25	11.40
**5d**	5.36	42.70
**5e**	6.05	52.80
**5f**	1.81	ND
**5g**	1.87	4.70
**5h**	1.27	2.50
**5i**	1.66	3.60
**5j**	1.18	12.70
**5k**	1.19	3.40
**5l**	1.67	6.60
**5m**	1.48	2.20
**5n**	1.77	4.50
**5o**	2.77	21.10
**5p**	1.82	6.60
**5q**	2.52	16.80
**5r**	2.13	11.70
**6b**	1.81	2.45
**6c**	2.28	11.60
**6d**	5.49	42.70
**6p**	1.77	6.80
**6q**	2.64	32.10
Fluorescein	2.55	23.80
Sulforhodamine B	1.52	27.80

Further experiments revealed that the synthesized dyes of type **2** and **5** as well as their NHS esters **3** and **6** were photostable—no changes were observed in the MS spectra of the water solutions of the dyes and only a minor decrease in the intensity of fluorescence was noted after 24 h irradiation with UV lamp (wavelength of 254 or 365 nm, 8 watts). Moreover, dyes **2** and **5** were stable in a broad range of pH (1–10), while their *N*-hydroxysuccinimide derivatives **3** and **6** proved stable under acidic, neutral, and slightly basic conditions. As expected, in alkaline pH, the reactive esters tend to be susceptible to hydrolysis and convert back into the free carboxylic acid forms **2** and **5**, respectively, as evidenced by MS analysis (data not shown).

### Fluorescence microscopy of prokaryotic cells

**Figure 2:**
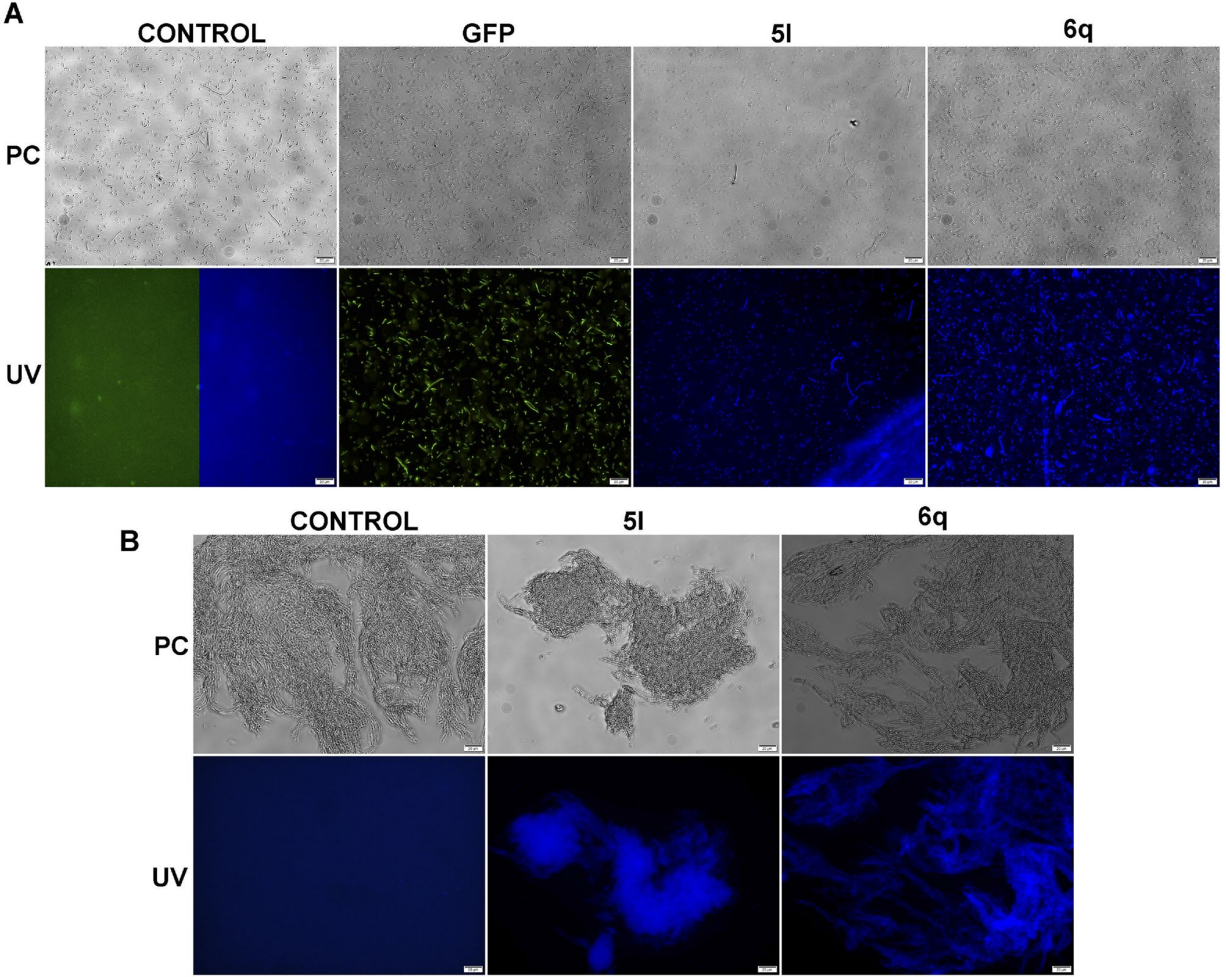
Phase contrast and fluorescence microscopy visualization of non-permebilized: (**A**) free floating *E. coli* and (**B**) aggregates of *B. mycoides*. Control—*E. coli* BL21DE3 and *B. mycoides* incubated without dyes, GFP—*E. coli* BL21DE3 producing GFP protein, **5l** and **6q**—*E. coli* BL21DE3 and *B. mycoides* stained with the appropriate dye, PC—phase-contrast microscopy and UV—fluorescence microscopy. Scale bars correspond to 20 µm.

All the synthesized dyes exhibited blue fluorescence (Supplementary Table S2). The applicability of *Safirinum* derivatives: **2a**,**d**, **3d**, **5c**,**l** and **6q** for one-step non-specific labeling of Gram-negative *E. coli* and Gram-positive *B. mycoides* bacteria has been tested. In the experiment, bacteria were simply incubated 105 min in phosphate buffered solution (PBS) with the appropriate dyes at a concentration of 2.5 mg/mL and, after washing out the non-bound compounds, analyzed by fluorescence microscopy. The compounds **2a**,**d** and **5c** stained both bacteria not-efficiently producing a weak fluorescence signal merging with background fluorescence for image acquisition times greater than 1 s (Supplementary Figs. S12 and S13). For the dyes **3d**, **5l** and **6q** the fluorescence signals of stained bacteria were effective even for 0.2 s of acquisition and their intensities were comparable to the signal of GFP-stained cells (Fig. 2, Supplementary Figs. S12, S13). It should be noted however that the fluorescence emitted by the bacteria stained with dye **3d** disappears within seconds, making the compound unsuitable for fluorescence microscopy. The fluorescence of bacteria stained with dyes **5l** and **6q** was stable after 24 h of incubation at 4 °C with a signal intensity reduction of ca. 20%. The staining effect with **3d**, **5l** and **6q** dyes of non-permeabilized and 70% isopropanol permeabilized *E. coli* and *B. mycoides* cells was almost identical, proving that these compounds do not require membrane permeabilization to effectively stain Gram-negative and Gram-positive bacteria, respectively. The dyes **5l** and **6q** were also effective for fluorescence staining of aggregates of *B. mycoides*, demonstrating their ability to penetrate the complex cells structures (Fig. 2).

### Fluorescence microscopy of eukaryotic cells

**Figure 3:**
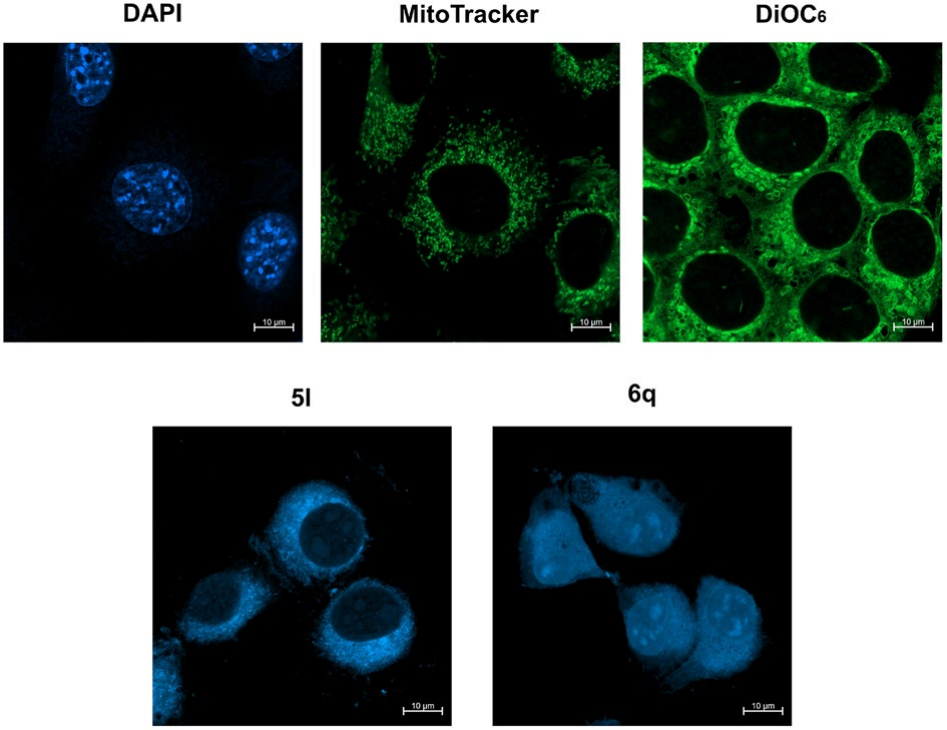
The staining effects of HEK293 cells: application of the tested dyes **5l** and **6q** as well as DAPI, MitoTracker Green and DiOC6 as controls. Scale bars correspond to 10 µm.

The next stage of our study was focused on testing 6 fluorescence dyes (**2a**,**d**, **3d**, **5c**, **5l** and **6q**) at a concentration of 2.5 mg/mL with respect to eukaryotic cells. The *Safirinium* derivatives were examined for potential application in visualization of cellular organelles by means of confocal microscopy. In this experiment, HEK293 cells we used and the slides were prepared in accordance with the described procedure. On the basis of the obtained results, it was observed that compounds **2a**,**d** and **5c** produced weak fluorescence signals (Supplementary Figs. S14 and S15). On contrary, the dye **3d** gave fluorescence signal effective even for 0.1 s, however, the long-lasting staining of the test samples was not attainable due to quick fading of the dye (after approx. 15 s). In the case of the dyes **5l** and **6q** the fluorescence signals of the stained cells were effective and quick fading of the dyes were not evidenced. The staining effects with all of the dyes tested on non-permeabilized and permeabilized cells were almost identical, indicating that these compounds do not require an increased membrane permeability to stain effectively. Next, we decided to elucidate the exact subcellular localization of the **5l** and **6q** dyes. Hence, in order to label the organelle the following fluorescent dyes have been utilized: MitoTracker Green dye to stain mitochondria, DiOC6 to stain mainly endoplasmatic reticulum and DAPI to stain cellular nuclei. It was found that the novel fluorescent dyes feature intracellular accumulation sites. Thus, compound **5l** targets mainly mitochondria and endoplasmic reticulum of cells, whilst the **6q** dye localizes primarily in cellular nuclei (Fig. 3).

### Fluorescent staining of human skin structures ex vivo

The studies of human skin and the ability to penetrate its outer layer by compounds such as cosmetic ingredients, xenobiotics or medicinal substances constitute a key aspect in medical, pharmaceutical, biological and biotechnological sciences. Microscopic techniques, including fluorescence microscopy, are valuable research methods that enable the imaging of biological samples, on the basis of which it is possible to observe and identify the structures as well as draw conclusions regarding the interactions of analyzed substances with building components of the skin, for example to assess the disorders of the *epidermis* layer after application of a substance to the skin. Fluorescence microscopy can provide information on the routes of penetration of a substance in various formulation forms and allow to diagnose skin diseases or to assess hair condition.

In the case of human skin imaging, the potential dyes must have the ability to diffuse deeply into the skin layer. The literature reports show that the penetration of the *stratum corneum*, which serves as a natural barrier, is conditioned by the diffusion process according to the first Fick's law. Molecules diffuse easily into the intercellular space composed of lipids when the molecular weight does not exceed 500 Da, log*P*_OW_ is in the range of 1–3, and the number of hydrogen bond acceptor (HBA) and hydrogen bond donor (HBD) is no more than 10 and 5, respectively^28–30^. An optimal dye should feature affinity to the skin structures, i.e. *stratum corneum*, which is built of corneocyte cells (composed of proteins—mainly keratin) and lipids of the lipid bilayer surrounding corneocytes (composed of various classes of ceramides, cholesterol esters, and free fatty acids). The most commonly used fluorescent dyes in the above context are fluorescein and rhodamine derivatives.

In the next step, 3 pyridine-based dyes (**2b**,**d** and **3d**) and 9 derivatives of quinoline (**5b–d**,**p**,**q** and **6b**,**c**,**p**,**q**) were used in skin staining experiments to select the compounds exhibiting desirable properties and the best staining effects. Derivatives with free carboxyl groups (**2b**,**d** and **5b–d**,**p**,**q**) as well as compounds with reactive (2,5-dioxopyrrolidin-1-yloxy)carbonyl (**3d** and **6b**,**c**,**p**,**q**) groups were utilized to verify the influence of structural modifications on cell structure visualization.

Fluorescent compounds were tested in two modes: by staining the control samples without application of the model substance and the test samples after application of the model substance, that is octamethylcyclotetrasiloxane (D4)—a siloxane with a cyclic structure.

### Stratum corneum staining efficiency of dyes with free carboxyl groups

**Figure 4:**
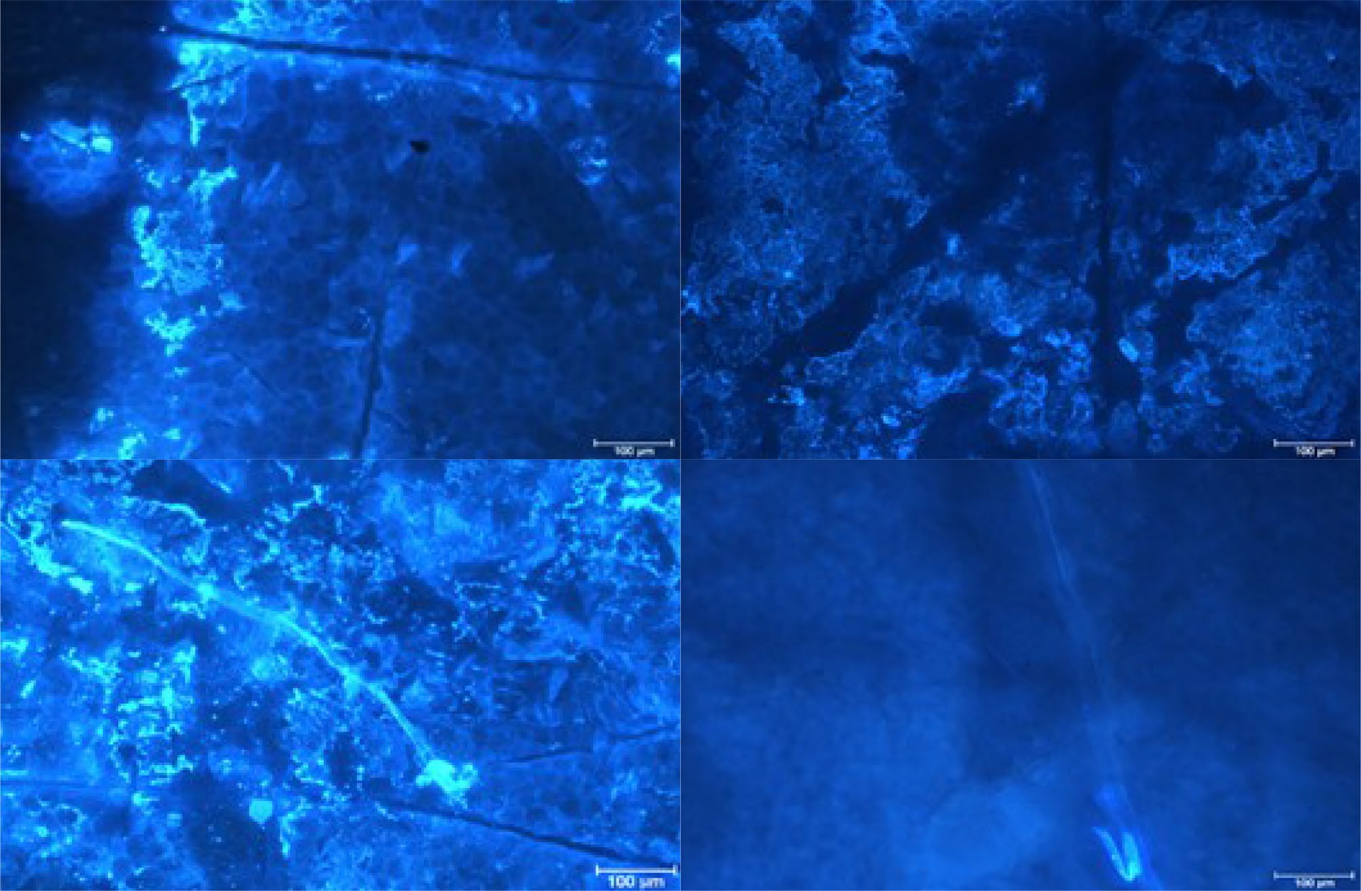
The staining effect of the samples: control (left panel) and tested (right panel) after application of dyes **5p** (upper panel) and **5q** (lower panel); magnification of microscopic images 10×.

On the basis of the obtained results, it was established that in the case of fluorescent compounds containing a carboxyl group on the pyridine ring (**2b**,**d**), the long-lasting staining of the control and the test samples was not attainable due to the quick fading of the dyes (after approx. 30 s). It was concluded that this effect could result from evaporation of the solvent from the surface of the formulations in which the fluorescent compounds were dissolved. In contrast, the quinoline derivatives (**5p**,**q**) retained longer fluorescent properties, although they did not prove effective ability to penetrate *stratum corneum* structures, staining mainly the surface of the skin (Fig. 4).

Further experiments proved that dyes with a free carboxyl group, and hence negatively ionized, do not diffuse into the *stratum corneum*, since no staining of the corneocytes and lipid matrix (the so-called lipid bilayer with lamellar structure) surrounding the corneocytes was observed. The obtained result is consistent with the mechanism of the skin barrier crossing described in the scientific literature, which assumes that ionized compounds feature much lower permeation coefficients than non-ionized compounds. Thus, proteins such as keratin present in corneocytes are ionized under physiological conditions presenting both positively and negatively charged fragments. For example, acidic proteins feature negatively charged amino acid side chains incorporating aspartic or glutamic acids, while basic proteins prove positively charged side chains build from lysine, arginine or histidine^31^. On the contrary, lipids bear negative charges, therefore it is generally assumed that the skin can act as a negatively charged membrane^31,32^. Consequently, electrostatic interactions may occur between the structural elements of the *stratum corneum* and ionized xenobiotics. The above results in repulsion of molecules (interaction of homonymous charges, e.g. negative ion-negative ion), which makes overcoming the skin barrier difficult or impossible. Thus, on the basis of the obtained results, it can be concluded that the negative charges located on the carboxylate groups (COO^-^) in the fluorescent dyes **2b**,**d** and **5p**,**q** condition poor or prevent penetration into the structures of *stratum corneum*. Consequently, positive charges on nitrogen atoms are sterically hindered making the COO^-^ groups exposed to electrostatic interactions with the negatively charged lipids of the lipid bilayer. This observations can be further supported by retention parameters obtained in the HPLC experiments. Hence, albeit log*k*_w_ values of the examined dyes **2b**,**d** and **5p**,**q** (1.13, 1.57, 1.82, 2.52, respectively) are comparable the corresponding parameters assessed for the reference fluorescein and sulforhodamine B (2.55, 1.52, respectively), the latter proved considerable higher affinities to phospholipids in CHI_IAM_ chromatography than the tested *Safirinium* dyes with (phospho)lipophilicity indices of 23.80 and 27.80 vs ND, 2.50, 6.60 and 16.80, respectively (Table 1). This observation stays in agreement with our previous study of *Safirinium–Q* hybrids, which despite having similar RP-HPLC lipophilicity indices to fluoroquinolone antibiotics, present significantly lower affinities to immobilized artificial membranes in IAM-HPLC analyses^21^.

### Stratum corneum staining efficiency with reactive ester dyes (3d and 6p)

**Figure 5:**
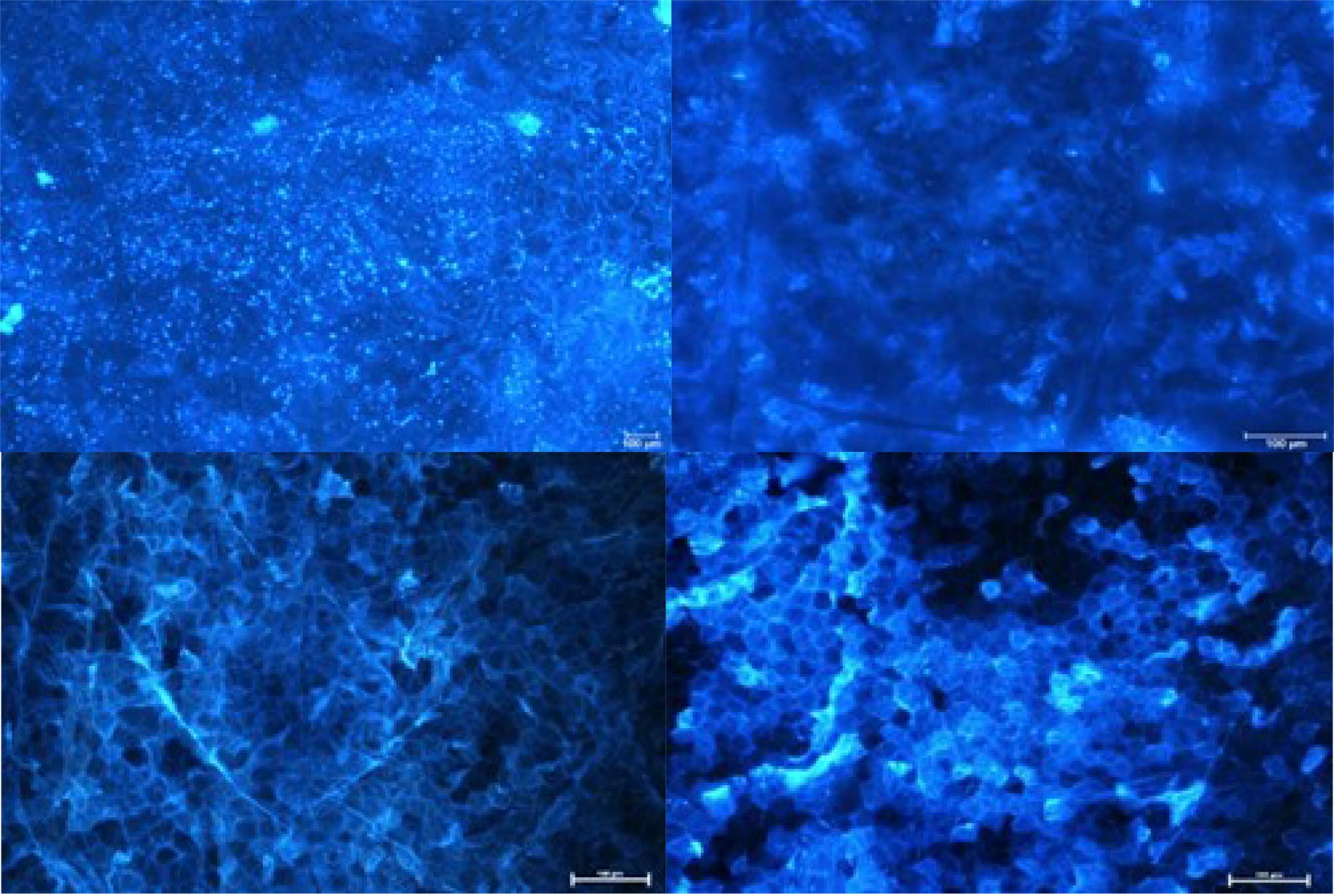
The effect of staining the samples: control (left panel) and tested (right panel) after applying the dyes containing the pyridine **3d** (upper panel) and a quinoline systems **6p** (lower panel); magnification of microscopic images 10×.

The next stage covered the assessment of *N*-hydroxysuccinimide esters of triazolopyridinium-carboxylate (**3d**) and triazoloquinolinium-carboxylate (**6p**) in terms of their utilization in imaging the *stratum corneum* structures. These two compounds were selected, taking into account their HPLC lipophilicity indices of 1.71 and 1.77, respectively, that correspond to the lipophilicity parameters assessed for the reference fluorescein (2.55) and sulforhodamine B (1.52). The conducted experiments has shown that compound **6p** of quinoline system and ethyl substituents within the triazolinium ring displays favorable fluorescent and diffusion properties to the skin in relation to the compound **3d** based on dibutyl-triazolopyridinium system (Fig. 5). Hence, in both the control and test samples (Fig. 5, lower panel) the corneocytes of pentagonal and hexagonal shape, typical for these cells, can be observed, which is not evidenced for samples stained with compound **3d** (Fig. 5, upper panel). Since both the discussed *N*-hydroxysuccinimide esters are of similar C_18_-HPLC lipophilic character the qualitative difference in their staining properties must result from other factors. Indeed, compound **6p** feature significantly lower affinity in to immobilized artificial membrane (CHI_IAM_ = 6.80) than ester **3d** (37.10), fluorescein (23.80) and sulforhodamine B (27.80). It should be pointed out that the dye **5p** featuring free carboxyl group (log*k*_w_ 1.82 and CHI_IAM_ = 6.60), despite having analogical physicochemical parameters to ester **6p** (log*k*_w_ 1.77 and CHI_IAM_ = 6.80), does not stain corneocytes within *stratum corneum* structures. Moreover, it was noted that application of the model liquid substance D4 to the skin increases of fluorescence intensity, which can be related to the solubility of the applied lipophilic dye (**6p**) in the model lipophilic substance (log*P* = 6.98; MW = 296.61 Da). In conclusion, it was found that the reactive (2,5-dioxopyrrolidin-1-yloxy)carbonyl moiety within the dye structure is crucial for visualization of corneocytes since it enables diffusion of the fluorescent dye into the *stratum corneum*.

### Examination of the effects alkyl substituents within the [1,2,4]triazolo[4,3-a]quinolin-2-ium system on the imaging efficiency of stratum corneum structures

The visualization efficiency of *stratum corneum* structures has been evaluated in relation to alkyl substitution pattern that condition lipophilicity of the *N*-hydroxysuccinimide esters of *Safirinium Q* dyes. Hence, compounds **6b**, **6c**, **6p** and **6q** of log*k*_w_ and CHI-_IAM_ parameters in the ranges 1.77 to 2.64 and 2.45 to 32.10, respectively, have been investigated (Table 2). From comparison of the obtained results, it can be concluded that compound **6b** devoid of methyl group within the quinoline ring features insufficient lipophilicity, which hinders its diffusion into the *stratum corneum*. In this case, the images were blurred, making it difficult to observe the corneocytes. In contrast, valuable microscopic images were obtained in other cases, indicating that the methyl group does not play a specific role in the staining of the structures, but has a significant effect in modifying the optimal lipophilicity of the compounds. In particular, the comparison of dibutyl derivatives **6c** and **6q**, where the compounds differ only in the presence or absence of a methyl group, indicates that efficient staining of *stratum corneum* must involve certain affinity to phospholipids (CHI-_IAM_ ≥ 6.80).

**Table 2:**
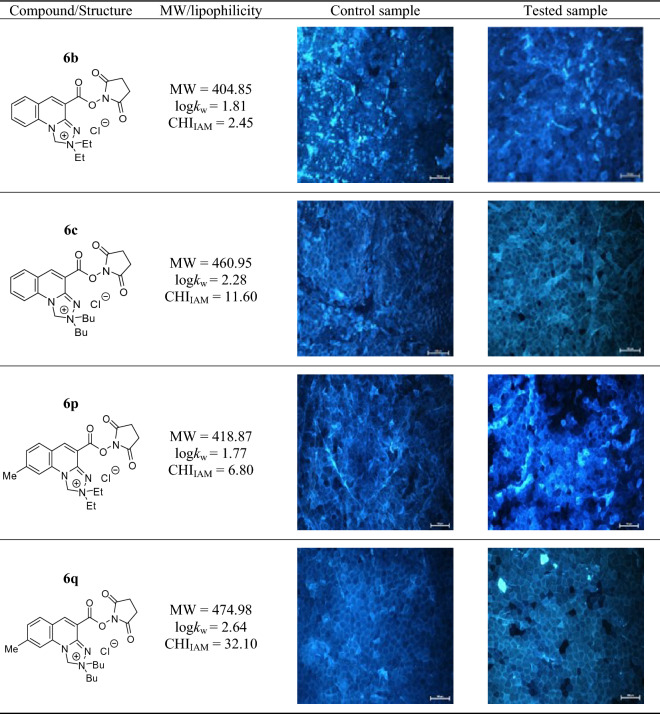
*Stratum corneum* structures stained with *N*-hydroxysuccinimide esters **6b**, **6c**, **6p** and **6q**; magnification of microscopic images 10x.

### Examination of the usefulness of Safirinium dyes for visualization of stratum corneum structures and its disorders under the influence of the model substance D4, as well as identification of the route of its penetration into the skin

As a result of the targeted modification of the *Safirinium Q* chromophore two novel and one known *N*-hydroxysuccinimide esters useful for visualization of *stratum corneum* structures were selected:

1. 2,2-dibutyl-4-((2,5-dioxopyrrolidin-1-yloxy)carbonyl)-1,2-dihydro-[1,2,4]triazolo[4,3-*a*]quinolin-2-ium chloride (**6c**);
2. 2,2-diethyl-4-((2,5-dioxopyrrolidin-1-yloxy)carbonyl)-8-methyl-1,2-dihydro-[1,2,4]triazolo[4,3-*a*]quinolin-2-ium chloride (**6p**);
3. 2,2-dibutyl-4-((2,5-dioxopyrrolidin-1-yloxy)carbonyl)-8-methyl-1,2-dihydro-[1,2,4]triazolo[4,3-*a*]quinolin-2-ium chloride (**6q)**.

**Figure 6:**
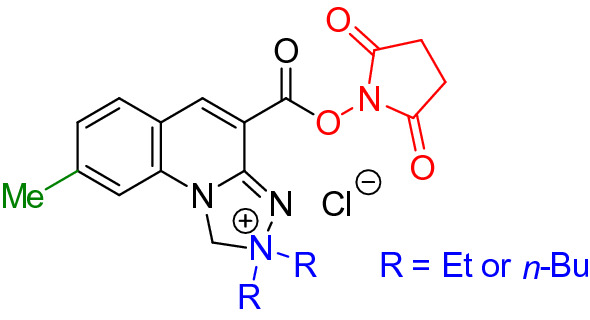
The regions of targeted modification of *Safirinium Q* chromophore essential for effective staining the *stratum corneum* structures.

The key modifications of the dyes with an estimated molecular weight lower than 500 Da included the introduction of:

- ethyl or butyl substituents at the N2 nitrogen atom (blue labeling);
- methyl substituent in the quinoline ring (green labeling);
- (2,5-dioxopyrrolidin-1-yloxo) moiety (red labeling) (Fig. 6).

**Figure 7:**
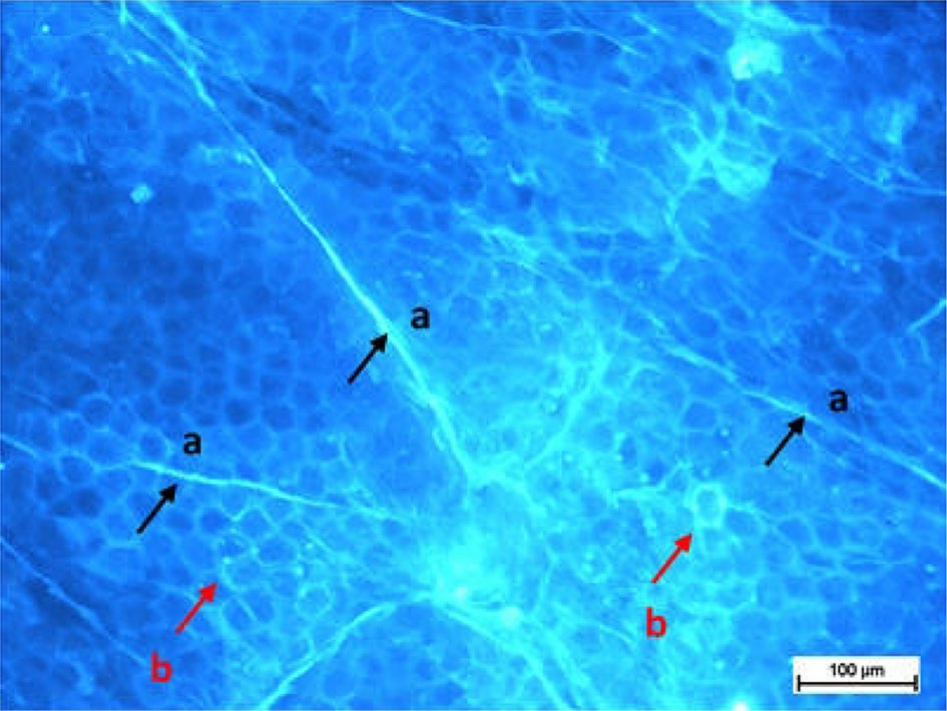
Exemplary microscopic image showing the transport pathways of the test substance (siloxane D4) into the skin via: (**a**) canyons (black arrows) and (**b**) lipids of the lipid bilayer (red arrows).

Application of the developed fluorescent dyes enabled effective microscopic visualization of the *stratum corneum* structures (Tables 3 and 4). In the control samples (Table 3, 40× and 100× magnification), corneocyte clusters of pentagonal or hexagonal shapes and a size of about 30–40 µm can be seen, which form a compact and tight structure. Moreover, inside the corneocytes keratin filaments could be observed, which are the main building blocks of these cells. Importantly, the lipid bilayer surrounding the corneocytes has been also stained. The results obtained indicate that the developed fluorescent dyes feature not only optimal lipophilicity conditioning diffusion into the skin, but also amphiphilic properties that determine affinity to lipophilic or hydrophobic structures, as well as hydrophilic and lipophobic ones. Thus, (a) lipophilic and hydrophobic structures include lipids (various classes of ceramides, cholesterol and its esters, free fatty acids, triglycerides) of which the lipid bilayer or corneocyte lipid envelope (CLE) is composed, while (b) hydrophilic and hydrophobic structures involve proteins (e.g., keratin, filaggrin, involucrin, loricrin, trichohyalin), which make up the corneocyte or cornified cell envelope (CCE). However, in the case of the test samples (Table 3) it was possible to observe the disturbed structure of corneocytes under the influence of the lipophilic test substance, which was siloxane of cyclic D4 structure. Hence, corneocytes lost their characteristic hexagonal or pentagonal shape (Table 4; 40× and 100× magnification), which resulted in the occurrence of so-called lacunae. In addition, the developed fluorescent dyes allowed identification of the transport pathways of D4 siloxane into the skin. Figure 7 shows that the test substance is distributed within the stained fluorescent lipid canyons, which surround clusters of *stratum corneum* corneocytes. The intensively colored canyons indicate that they constitute an important penetration pathway for the lipophilic xenobiotics into the skin. It was also demonstrated that compound D4 diffuses into the *stratum corneum* via transepidermal transport through the lipid matrix, which was confirmed by intensely stained lipid structures localized around the corneocytes.

**Table 3:**
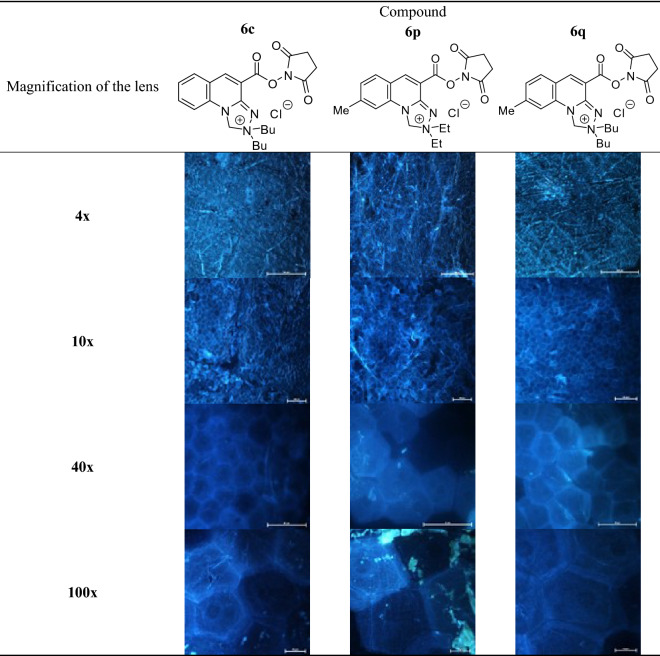
Visualization of the *stratum corneum* structures in the control samples stained with the optimized *Safirinium* dyes (**6c**, **6p** and **6q**).

**Table 4:**
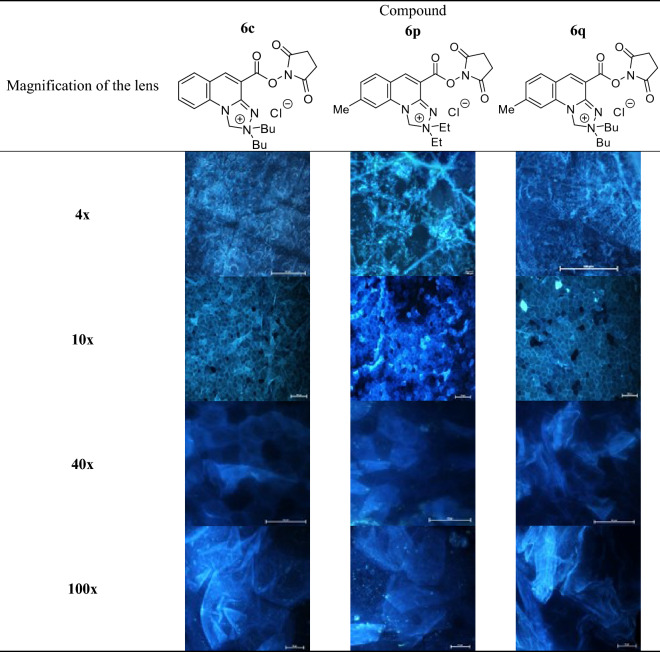
Visualization of the *stratum corneum* structures in the samples treated with siloxane D4 and stained with the optimized *Safirinium* dyes (**6c**, **6p** and **6q**).

## Conclusions

The presented study was focused on the targeted chemical modifications of *Safirinium* dyes in the perspective of their applications in imaging of biological structures. It has been proved that the obtained compounds exhibit fluorescent properties in diverse biological systems and facilitate visualization of various organisms including Gram-positive and negative bacteria, human cell line, as well as *stratum corneum* tissue crucial for barrier function of the skin. The advantageous features of the obtained compounds include inexpensive synthesis and molecular weight below 500 Da, which is especially important in labeling of small structures such as lambda phages^33^. These properties could render *Safirinium* dyes superior to large and expensive commercially available dyes. Moderately lipophilic *N*-hydroxysuccinimide ester of *Safirinium Q* dye (**6q**) was found to be effective in staining nuclei of human kidney cells, while compounds **5l** and **6q** were successfully applied in labeling of non-permeabilized and permeabilized *E. coli* and *B. mycoides* cells as well as mitochondria and endoplasmic reticulum of eukaryotic HEK293 cells. *Safirinium Q* analogues with NHS moiety (**6c**, **6p** and **6q**) were found to be effective in visualization of horny layer of the *epidermis*, i.e. corneocytes, lipid multilamellae region surrounding these cells as well canyons surrounding the clusters of the corneocytes. The dyes were found also applicable in evaluation of siloxane exposure and xenobiotic penetration into the skin. The staining effectiveness of the developed dyes has been associated with their lipophilicity and phospholipophilicity parameters assessed in the corresponding HPLC analyzes.

## Methods

### General information

All chemicals were used as received from commercial sources (Acros Organics — Geel, Belgium; Alfa-Aesar—Ward Hill, MA, USA; Sigma-Aldrich — Steinheim, Germany; or Polish Chemical Reagents—Gliwice, Poland) and used without further purification. Distilled water was obtained from System Elix 3 (Millipore, Bedford, USA). All nuclear magnetic resonance (NMR) spectra were recorded on a Varian Mercury-VX 300 MHz, Bruker Avance III HD 400 MHz, or Varian Unity Plus 500 MHz spectrometers at 25 °C. Chemical shifts (δ) are given in parts per million (ppm) and internally referenced to CD_3_OD (^1^H: δ = 3.31 ppm; ^13^C: δ = 49.00 ppm) or DMSO-*d*_*6*_ (^1^H: δ = 2.50 ppm) signals. Splitting patterns are designated as s (singlet), bs (broad singlet), d (doublet), t (triplet), or m (multiplet), the coupling constants (*J*) are reported in Hertz (Hz). The IR (KBr pellets) spectra were recorded on a Thermo Scientific Nicolet 380 FT-IR spectrometer. Melting points were determined on an X-4 melting point apparatus with a microscope and were uncorrected. The mass spectra were recorded on single quadrupole LCMS 2010 EV (Shimadzu—Kyoto, Japan) mass spectrometer.

### Chemical synthesis

Proflurophores **1** and **4a-g**, *Safirinium P* (**2a–c**,**e–h**) and *Q* (**5a,b**,**d–p**,**r**) dyes, as well as their reactive NHS esters **3b,e** and **6b**,**d**,**q** were characterized previously by our research group and synthesized according to the reported procedures^14,15,17,18,34^.

### Synthesis of Safirinium P (2d) and Q (5c,q) derivatives

2,2-Dibutyl analogues of dihydro-[1,2,4]triazolo[4,3-*a*]pyridin-2-ium-8-carboxylate (**2d**) and dihydro-[1,2,4]triazolo[4,3-*a*]quinolin-2-ium-4-carboxylates (**5c**,**q**) were obtained from the corresponding isoxazolones (1.9 mmol of **1** and **4a**,**f**, respectively) dissolved in methanol (10 mL). Dibutylamine (0.19 mL, 1.9 mmol) and formaldehyde 35 wt. % aqueous solution (0.6 mL, 7.6 mmol) were added to the solution and the resulting mixture was stirred at room temperature for 16 h. The progress of the reaction was monitored by LC–MS. The reaction mixture was evaporated under reduced pressure and subsequently, the crude product was washed with acetone (2 × 3 mL).

### 2,2-Dibutyl-5,7-dimethyl-2,3-dihydro-[1,2,4]triazolo[4,3-a]pyridin-2-ium-8-carboxylate (2d)

Obtained from 4,6-dimethylisoxazolo[3,4-*b*]pyridin-3(1*H*)-one **1** (0.312 g). Yield: 83% (0.481 g); m.p. 139–141 °C; ^1^H NMR (300 MHz, CD_3_OD): δ = 0.99 (t, *J* = 7.4 Hz, 6H, CH_3_), 1.35–1.47 (m, 4H, CH_2_), 1.74–1.84 (m, 4H, CH_2_), 2.20 (s, 3H, CH_3_), 2.30 (s, 3H, CH_3_), 3.45–3.62 (m, 4H, CH_2_), 5.72 (s, 2H, CH_2_), 5.99 (s, 1H, CH); ^13^C NMR (75 MHz, CD_3_OD): δ = 12.6, 16.9, 18.4, 19.2, 24.1, 66.2, 72.9, 110.1, 118.0, 139.1, 146.2, 155.6, 170.2; IR (KBr): 3394, 3001, 2962, 2983, 2871, 1668, 1634, 1619, 1560, 1529, 1467, 1374, 1356, 1188, 801, 606 cm^−1^; MS (ESI) m/z: 306 [M + 1]^+^.

### 2,2-Dibutyl-1,2-dihydro-[1,2,4]triazolo[4,3-a]quinolin-2-ium-4-carboxylate (5c)

Obtained from isoxazolo[3,4-*b*]quinolin-3(1*H*)-one **4a** (0.354 g). Yield: 98% (0.610 g); m.p. 176–179 °C; ^1^H NMR (300 MHz, CD_3_OD): δ = 0.98 (t, *J* = 7.3 Hz, 6H, CH_3_), 1.37–1.49 (m, 4H, CH_2_), 1.76–1.87 (m, 4H, CH_2_), 3.60–3.77 (m, 4H, CH_2_), 5.84 (s, 2H, CH_2_), 7.18 (d, *J* = 7.8 Hz, 1H, CH), 7.32 (t, *J* = 7.8 Hz, 1H, CH), 7.66 (t, *J* = 7.8 Hz, 1H, CH), 7.74 (d, *J* = 7.8 Hz, 1H, CH), 8.18 (s, 1H, CH); ^13^C NMR (75 MHz, CD_3_OD): δ = 12.6, 19.2, 24.2, 66.1, 71.8, 113.2, 121.0, 121.9, 123.4, 129.9, 132.4, 134.3, 140.9, 154.9, 167.8; IR (KBr): 3401, 2962, 2934, 2875, 1612, 1588, 1569, 1540, 1457, 1365, 1348, 1297, 1216, 809, 762, 594, 528 cm^-1^; MS (ESI) m/z: 328 [M + 1]^+^.

### 2,2-Dibutyl-8-methyl-1,2-dihydro-[1,2,4]triazolo[4,3-a]quinolin-2-ium-4-carboxylate (5q)

Obtained from 7-methylisoxazolo[3,4-*b*]quinolin-3(1*H*)-one **4f.** (0.380 g). Yield: 91% (0.590 g); m.p. 230–235 °C; ^1^H NMR (400 MHz, CD_3_OD): δ = 1.02 (t, *J* = 7.5 Hz, 6H, CH_3_), 1.45–1.50 (m, 4H, CH_2_), 1.80–1.89 (m, 4H, CH_2_), 2.56 (s, 3H, CH_3_), 3.67–3.79 (m, 4H, CH_2_), 5.88 (s, 2H, CH_2_), 7.11 (s, 1H, CH), 7.28 (d, *J* = 8.1 Hz, 1H, CH), 7.79 (d, *J* = 8.1 Hz, 1H, CH), 8.71 (s, 1H, CH); ^13^C NMR (100 MHz, CDCl_3_): δ = 12.6, 19.2, 21.0, 24.2, 66.3, 71.8, 111.9, 114.0, 118.1, 125.4, 130.9, 135.7, 146.8, 147.1, 154.1, 163.5; IR (KBr): 3422, 3003, 2962, 2978, 2897, 2874, 1721, 1616, 1555, 1221, 1199, 1182, 1159, 1119, 816, 736 cm^-1^; MS (ESI) m/z: 342 [M + 1]^+^.

### Synthesis of 2,2-dibutyl Safirinium P (3d) and Q (6c,q) N-hydroxysuccinimide esters

Dibutyl deriatives of 8-((2,5-dioxopyrrolidin-1-yloxy)carbonyl)-5,7-dimethyl-2,3-dihydro-[1,2,4]triazolo[4,3-*a*]pyridin-2-ium (**3d**) and 4-((2,5-dioxopyrrolidin-1-yloxy)carbonyl)-1,2-dihydro-[1,2,4]triazolo[4,3-*a*]quinolin-2-ium (**6c**,**q**) chlorides were obtained according to literature procedures reported earlier by our research group^14^ with only minor modifications. The compounds **2d** and **5c**,**q** (0.35 mmol) obtained in zwitterionic forms were dissolved in methanol and acified with methanolic HCl solution to get hydrochlorides **2d** and **5c**,**q**. The solvent was evaporated under reduced pressure, then the resulted solids were dissolved in anhydrous DMF (7 mL). Subsequently, NHS (0.060 g, 0.525 mmol) and (DIC) (0.082 mL, 0.525 mmol) were added and the reaction mixtures were stirred for 2–4 h at room temperature. The progress of the reaction was monitored by LC–MS. After the completion of the reaction, the solvent was evaporated and the crude product was isolated. Compound **2d** was dissolved in water (5 mL) and filtered, then the filtrate was evaporated. The resulted solid was washed with acetone (3 × 2 mL) and dryied under vacuum. The compounds **5c**,**q** were washed with acetone (3 × 2 mL) and dried, then purified by crystallization from methanol/aceton mixture (1:20, v/v).

### 2,2-Dibutyl-8-((2,5-dioxopyrrolidin-1-yloxy)carbonyl)-5,7-dimethyl-2,3-dihydro-[1,2,4]triazolo[4,3-a]pyridin-2-ium chloride (3d)

Obtained from compound **2a** (0.107 g). Yield: 48% (0.074 g); m.p. 150–151 °C; ^1^H NMR (500 MHz, DMSO-*d*_*6*_): δ = 0.92 (t, *J* = 7.3 Hz, 6H, CH_3_), 1.33–1.35 (m, 4H, CH_2_), 1.61–1.82 (m, 4H, CH_2_), 2.40 (s, 3H, CH_3_), 2.45 (s, 3H, CH_3_), 2.88 (s, 4H, CH_2_), 3.61–3.67 (m, 4H, CH_2_), 5.84 (s, 2H, CH_2_), 6.31 (s, 1H, CH); IR (KBr): 3416, 2963, 2929, 2874, 1776, 1739, 1626, 1562, 1520, 1368, 1206, 1139, 1114, 1062, 994, 984, 646 cm^−1^; MS (ESI) m/z: 403 [M]^+^.

### 2,2-Dibutyl-4-((2,5-dioxopyrrolidin-1-yloxy)carbonyl)-1,2-dihydro-[1,2,4]triazolo[4,3-a]quinolin-2-ium chloride (6c)

Obtained from compound **5c** (0.115 g). Yield: 43% (0.069 g); m.p. 168–169 °C; ^1^H NMR (500 MHz, DMSO-*d*_*6*_): δ = 0.93 (t, *J* = 7.4 Hz, 6H, CH_3_), 1.34–1.48 (m, 4H, CH_2_), 1.70–1.83 (m, 4H, CH_2_), 2.93 (s, 4H, CH_2_), 3.68–3.96 (m, 4H, CH_2_), 5.94 (s, 2H, CH_2_), 7.26 (d, *J* = 7.9 Hz, 1H, CH), 7.43 (t, *J* = 7.9 Hz, 1H, CH), 7.92 (t, *J* = 7.9 Hz, 1H, CH), 8.12 (d, *J* = 7.9 Hz, 1H, CH), 9.11 (s, 1H, CH); IR (KBr): 3341, 2967, 2935, 2876, 1774, 1738, 1718, 1619, 1572, 1459, 1362, 1246, 1202, 1168, 1138, 1093, 1070, 996, 765, 665 cm^-1^; MS (ESI) m/z: 425 [M + 1]^+^.

### 2,2-Dibutyl-4-((2,5-dioxopyrrolidin-1-yloxy)carbonyl)-8-methyl-1,2-dihydro-[1,2,4]triazolo[4,3-a]quinolin-2-ium chloride (6q)

Obtained from compound **5q** (0.120 g). Yield: 96% (0.161 g); m.p. 199–202 °C; ^1^H NMR (500 MHz, DMSO-*d*_*6*_): δ = 0.93 (t, *J* = 7.3 Hz, 6H, CH_3_), 1.34–1.38 (m, 4H, CH_2_), 1.70–1.81 (m, 4H, CH_2_), 2.60 (s, 3H, CH_3_), 2.92 (s, 4H, CH_2_), 3.62–3.74 (m, 4H, CH_2_), 5.93 (s, 2H, CH_2_), 7.13 (s, 1H, CH), 7.28 (d, *J* = 8.2 Hz, 1H, CH), 8.00 (d, *J* = 8.2 Hz, 1H, CH), 9.05 (s, 1H, CH); IR (KBr): 3341, 3023, 2966, 2935, 2876, 1775, 1740, 1618, 1562, 1198, 1096, 1066, 920, 816, 765, 644 cm^-1^; MS (ESI) m/z: 439 [M]^+^.

### HPLC analysis

The determinations of lipophilicity and affinity to phospholipids were performed using chromatographic gradient approaches. Each chromatographic experiment was carried out using the Prominence-1 LC-2030C 3D HPLC system (Shimadzu, Japan) controlled by LabSolution system (version 5.90 Shimadzu, Japan). Detection of solutes was performed at 200 nm using DAD detector.

Organic solvents, i.e. acetonitrile and methanol (HPLC grade for liquid chromatography), sodium phosphate dibasic dehydrate, and sodium phosphate monobasic monohydrate were purchased from Sigma-Aldrich (Steinheim, Germany). Ultrapure water used for buffer mobile phase preparation was obtained with Millipore Direct-Q 3 UV Water Purification System (Millipore Corporation, Bedford, MA, USA).

The RP-HPLC analyses were performed on Knauer 100–5 C_18_ 4.6 × 150 HPLC column with a linear gradient 25–98% phase B (where phase A was water and phase B was methanol) with a flow rate of 1 mL/min. The temperature of the chromatographic column was controlled and set to 30.0 °C. Two gradient runs of diverse gradient time (t_G_ corresponds to 15 min. and 30 min.) were performed. The retention times (tR) of the investigated solutes are listed in Table A. These data were used as input values for log*k*_w_ calculation (i.e. the log*k* retention factor extrapolated to 0% organic modifier) using DryLab 6.0 software (Molnar Institute, Berlin, Germany), according to the theory proposed by Snyder and co-workers. The dwell volume of the HPLC system was 0.780 mL, whereas the measured dead time for the utilized HPLC columns was 1.401 min.

The IAM-HPLC analyses were carried on IAM.PC.DD2 column (10 × 4.6 mm; particle size 10.0 µm with IAM guard column; Regis Technologies, USA) with a linear gradient 0–85% phase B (where phase A was 10 mM phosphoric buffer at pH 7.4 and phase B was acetonitrile) at a flow rate of 1.5 mL/min and constant temperature of 30.0 °C. The standards of octanonophenone, butyrophenone, and acetanilidine were provided by Alfa Aesar (Haverhill, MA, USA); acetaminophen, theophylline, benzimidazole, acetophenone and indole were purchased from Sigma-Aldrich (Steinheim, Germany); heptanophenone, hexanophenone, valerophenone, propiophenone, acetophenone were acquired from Acros Organic (Massachusetts, United States). The reference substances were utilized to determine CHI indices of the investigated dyes according to the protocol proposed by Valkó and co-workers^35^. The chromatographic analyses were performed in duplicate.

### Fluorescence microscopy of bacteria

The following bacterial strains were used: *E. coli* BL21DE3 (Merck), *E. coli* BL21DE3 encoding pSFOXB20-daGFP plasmid (Oxford Genetics) for constitutive production of GFP (Green Fluorescent Protein) and *Bacillus mycoides* (ATCC 6462). *E. coli* and *B. mycoides* were cultivated overnight with agitation at 37 and 30 °C, respectively, in Luria broth (LB) supplemented with the kanamycin (20 μg/mL) for BL21DE3/pSFOXB20-daGFP strain. Overnight cultures were suspended in phosphate-buffered saline (PBS) to OD_600_ = 0.5. The cells were incubated at room temperature in 70% isopropanol for 15 min to prepare bacteria with a permeabilizded membrane. Then the permeabilized cells were washed with PBS and finally suspended in PBS buffer to OD_600_ = 0.5. *B. mycoides* aggregates were induced by incubation of bacteria at room temperature without shaking for 4 h in PBS. For fluorescence microscopy, bacteria suspended in PBS were incubated with **2a**,**d**, **3d**, **5c**,**l** and **6q** dyes at a final concentration of 2.5 mg/mL for 105 min at room temperature in the dark. Then, the non-bound dyes were removed by washing with PBS buffer. The *E. coli* and *B. mycoides* as permeabilized, non-permeabilized or aggregated cells were analyzed in suspension as a drop loaded on a slide glass using the Olympus IX73 inverted fluorescence microscope equipped with the UCPlanFL N 20x/0.70, a Olympus U-HGLGPS fluorescence light source and a Hamamatsu Orcaflesh 2.8 CMOS digital camera. For blue and green fluorescence the following filter sets were used: DAPI HC BP, GFP-1828A and TRITC-B-000 (all from Semrock, IDEX Corporation), respectively. Each type of experiment was repeated 3 times.

### Eukaryotic cells imaging

Human embryonic kidney cell line (HEK-293) from American Type Culture Collection (Manassas, USA) was used in this study. The cells were maintained in cell media consisting of Dulbecco’s modified Eagles medium (DMEM) (Corning, USA) with 10% fetal bovine serum (FBS), (Corning, South America), 1% penicillin/streptomycin (Sigma-Aldrich Inc., Ireland), and 1% L-glutamine (Corning, USA). HEK-293 cells were cultured in an incubator at 37 °C and 10% CO_2_.

In this study the cells were grown in slides placed in a 35 mm dish. After 24 h incubation the cells were washed with PBS × 1 and fixed by adding 1 mL per dish of a 4% paraformaldehyde solution for 15 min at room temperature. To prepare cells with a permeabilized membranes, the cells were incubated at room temperature in 0.2% PBS-Triton × 1100 for 10 min. After washing twice with PBS × 1, the cells were incubated for 105 min at room temperature in the dark with **2a**,**d**, **3d**, **5c**,**l** and **6q** dyes at final concentration of 2.5 mg/mL diluted with PBS × 1. Nuclei were stained in a 0.25 μg/mL 4′,6-diamino-2-phenylindole (DAPI) solution. MitoTracker Green at concentration of 500 nM was used to stain the mitochondria. 3,3′-Dihexyloxacarbocyanine iodide (DiOC_6_) was used for the staining of a cell's membranes including endoplasmic reticulum and vesicle membranes, at final concentration of 5 µM.

The slides were observed using the confocal microscope (Zeiss LSM 800) at magnifications of 63×, applying appropriate filters and wavelengths.

The cytotoxicity of the prepared compounds **2b** and **5b** was examined in human embryonic kidney 293 cells (HEK 293) using the 3-(4,5-dimethylthiazol-2-yl)-2,5-diphenyltetrazolium bromide (MTT) cell viability assay. No reducing trends to the cell viability or changes in cell morphology were observed (data not shown). All the tested compounds were found to be non-toxic up to the concentration of 20 µM.

### Preparation of the ex vivo human skin

Human cadaver skin was used, obtained during necropsy. Skin section (15–20 cm length, 2–3 cm width) from the abdominal region of males and females in the age group of 35–50 years was collected 48 h hours after the time of death. The study has been approved by the Independent Bioethics Commission for Research at the Medical University of Gdańsk (no NKBBN/309/2013 and no NKBBN/449/2020). After separation of subcutaneous tissue, skin was washed with purified water and 0.9% w/v NaCl solution, dried and fragmented into smaller pieces, protected with aluminium foil and stored in a freezer at −20 °C, labelled with the batch number (donor). Prior to examination, a section of skin was thawed. Then, skin sample integrity was examined by the Trans-Epithelial Electrical Resistance technique (TEER). Millicell ERS-2 (Millipore, USA) data bridge connected to two stainless steel electrodes was used; ER was expressed in kΩ for the surface area of the exposed skin (0.65 cm^2^). All measurements of skin samples demonstrated values above the limit of 2 kΩ/cm^2^ (average 5 kΩ/cm^2^), indicating the integrity of the skin^36–38^. After placing the fragment in a chamber, 10 µL of siloxane D4 was applied to the skin surface of the test sample (control sample—the same procedure but without application). After 24 h of incubation, the remaining siloxane was removed from the sample surface using successively: filter paper strips, a hexane-soaked swab, and by removing the top three layers of *stratum corneum* by tape-stripping technique with special adhesive tapes (Book Tape 845 Scotch® (3 M, USA). The e*pidermis* layer was then isolated from *dermis* using the heat-separation technique (65 °C, 40 s, manually using a forceps). Then, *epidermis* was washed with distilled water and cut into smaller pieces. Samples were next transferred to a microscope slide with the *stratum corneum* on top and dried for 2 h.

We declare that the *ex-vivo* studies were performed using human cadaver skin that is unidentified decedents examined at the Department of Forensic Medicine, Faculty of Medicine, Medical University of Gdańsk, Poland. The Independent Bioethics Commission for Research at the Medical University of Gdańsk waived the need for consent to harvest the human skin samples (no NKBBN/309/2013 and NKBBN/449/2020).

Microscopic images were recorded using a Nikon Eclipse 50 fluorescence microscope with a mercury lamp. 10 µL of the appropriate dye was topically applied to epidermal sample, omitting the pore areas left by the hair follicles. The sample was placed in a chamber consisting of two Petri dishes, lined with a piece of lignin, previously moistened with purified water. The incubation time at 32 °C ± 1 °C in a thermostat was 10 min. In the next step, the residual dye was gently removed from the sample surface with a piece of lignin. Next, samples were examined using the fluorescence microscope at magnifications of 4, 10, 40, and 100 x, applying appropriate filters and wavelengths. A series of images were taken at different depths of the sample (changing the position of the table by 0.5–1.0 µm average) with utilization of automatic z-axis drive. Image registration and processing was performed using "NIS Elements AR3.2" software.

## Supplementary Information

Supplementary Information.

## Data Availability

All data generated or analyzed during this study are included in this published article (and its Supplementary Information file). The following data are available: The NMR spectra (Supplementary Figs. S1–S8); MS spectra (Supplementary Figs. S9–S11); visualization of free floating non-permeabilized and permeabilized *E. coli* BL21DE3 and *B. mycoides* (Supplementary Figs. S12 and S13); microscopy visualization of HEK293 cells (Supplementary Figs. S14 and S15); the chromatographic retention data (Supplementary Table S1); wavelengths of maximum absorbance and emission (Supplementary Table S2).
