# Supplementary material for: Development of Safirinium dyes for new applications: fluorescent staining of bacteria, human kidney cells, and the horny layer of the epidermis
Source: Sci Rep. 2022 Sep 5;12:15098. doi: 10.1038/s41598-022-19262-w (PMC9445088; doi:10.1038/s41598-022-19262-w)
Supplement: Supplementary file 1 — Supplementary Information. [file 41598_2022_19262_MOESM1_ESM.docx]

**Supplementary Information**

**Development of *Safirinium* dyes for new applications – fluorescent staining of bacteria, human kidney cells, and horny layer of the *epidermis***

Joanna Fedorowicz^1,2,^*, Dagmara Bazar^3^, Wioletta Brankiewicz^4^, Hanna Kapica^3^, Krzesimir Ciura^3,5^, Beata Zalewska-Piątek^6^, Rafał Piątek^6,7^, Krzysztof Cal^8^, Krystyna Mojsiewicz-Pieńkowska^3^ and Jarosław Sączewski^9^

^1^Drug Research Program, Division of Pharmaceutical Biosciences, Faculty of Pharmacy, University of Helsinki, P.O. Box 56 (Viikinkaari 5 E), Helsinki, 00014, Finland

^2^Department of Chemical Technology of Drugs, Faculty of Pharmacy, Medical University of Gdańsk, Al. Gen. J. Hallera 107, 80-416 Gdańsk, Poland

^3^Department of Physical Chemistry, Medical University of Gdańsk, Al. Gen. Hallera 107, 80-416 Gdańsk, Poland

^4^Department of Pharmaceutical Technology and Biochemistry, Chemical Faculty, Gdańsk University of Technology, Narutowicza 11/12, 80-233 Gdańsk, Poland

^5^QSAR Lab Ltd., Trzy Lipy 3 St., Gdańsk, 80-172, Poland

^6^Department of Molecular Biotechnology and Microbiology, Chemical Faculty, Gdańsk University of Technology, Narutowicza 11/12, 80-233 Gdańsk, Poland

^7^BioTechMed Center, Gdańsk University of Technology, Narutowicza 11/12, 80-233 Gdańsk, Poland

^8^Department of Pharmaceutical Technology, Medical University of Gdańsk, Al. Gen. J. Hallera 107, 80-416 Gdańsk, Poland

^9^Department of Organic Chemistry, Faculty of Pharmacy, Medical University of Gdańsk, Al. Gen. J. Hallera 107, 80-416 Gdańsk, Poland

*Corresponding author.

E-mail address: jfedorowicz@gumed.edu.pl (J. Fedorowicz)

|  | **Table of contents:** | Page |
| --- | --- | --- |
| 1 | NMR spectra | 2 |
| 2 | Visualization of bacteria | 8 |
| 3 | Microscopy visualization of HEK293 cells | 10 |
| 4  5 | Retention factors  Wavelengths of maximum absorbance and emission | 12  13 |


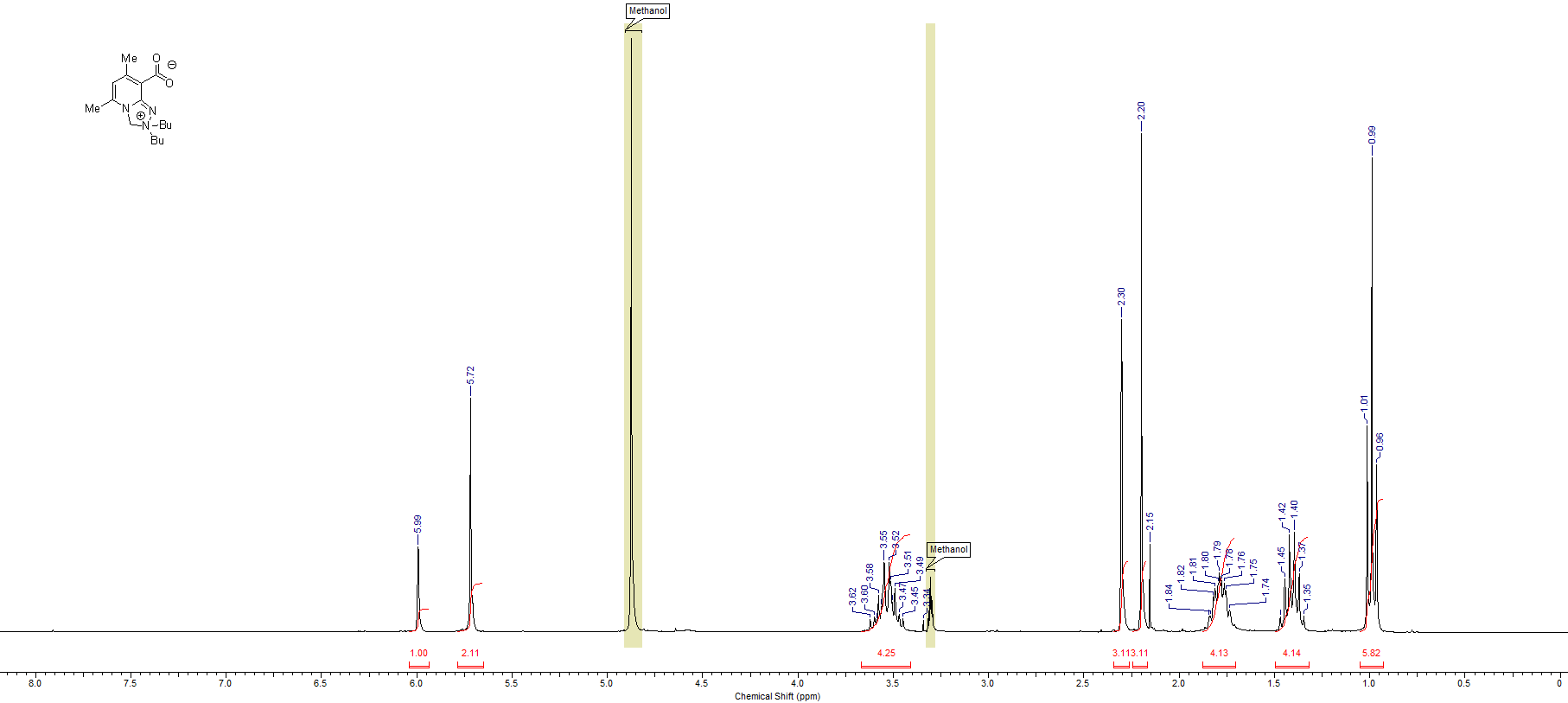


**Figure S1.** ^1^H-NMR (300 MHz, CD_3_OD) spectrum of 2,2-dibutyl-5,7-dimethyl-2,3-dihydro-[1,2,4]triazolo[4,3-*a*]pyridin-2-ium-8-carboxylate (**2d**).


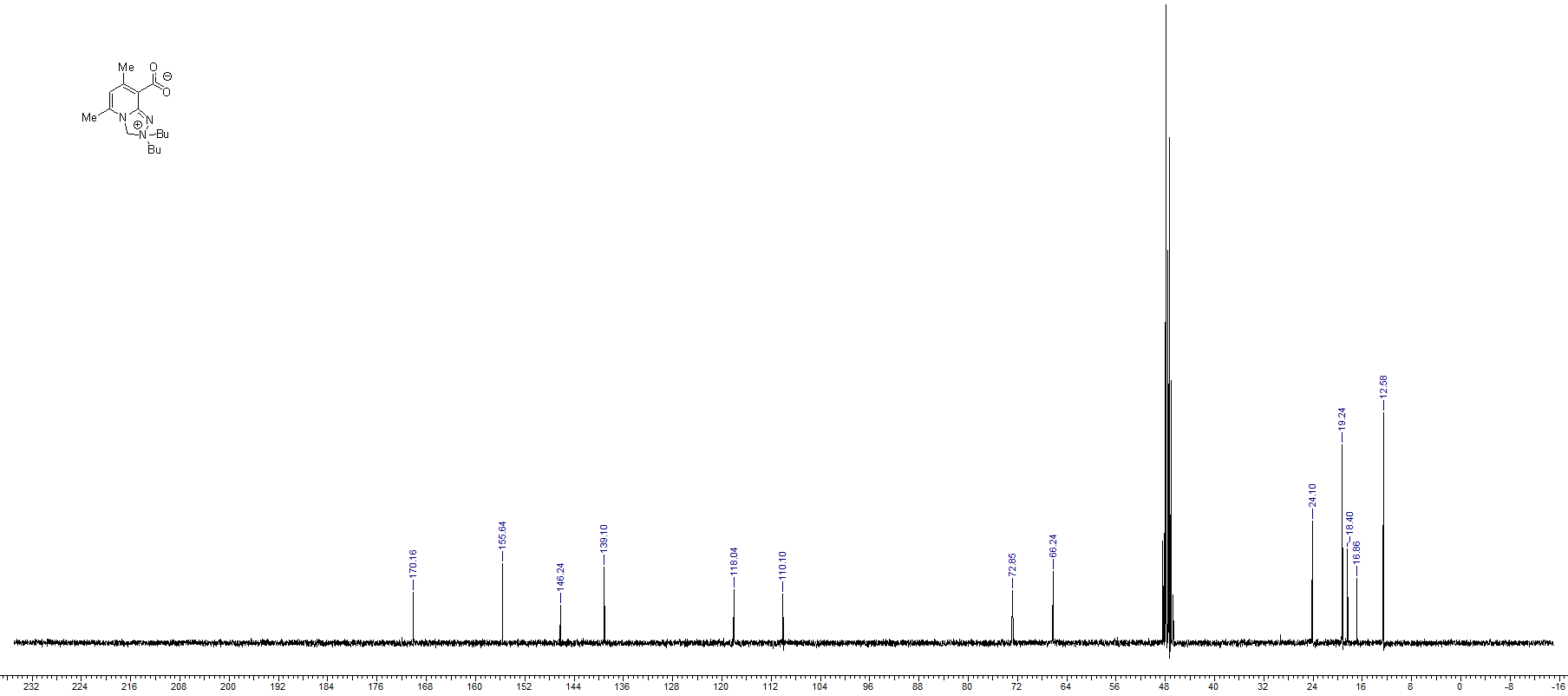


**Figure S2.** ^13^C-NMR (75 MHz, CD_3_OD) spectrum of 2,2-dibutyl-5,7-dimethyl-2,3-dihydro-[1,2,4]triazolo[4,3-*a*]pyridin-2-ium-8-carboxylate (**2d**).


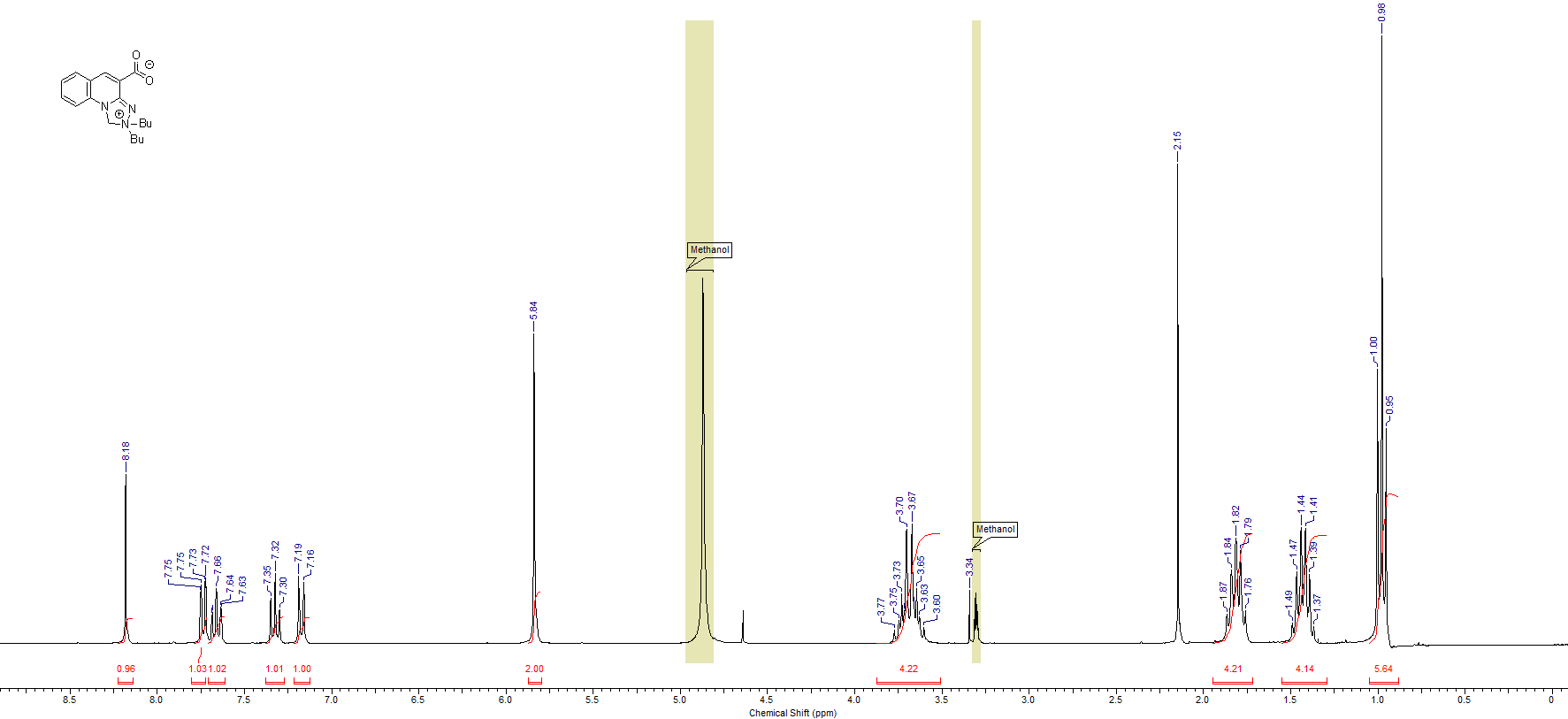


**Figure S3.** ^1^H-NMR (300 MHz, CD_3_OD) spectrum of 2,2-dibutyl-1,2-dihydro-[1,2,4]triazolo[4,3-*a*]quinolin-2-ium-4-carboxylate (**5c**).


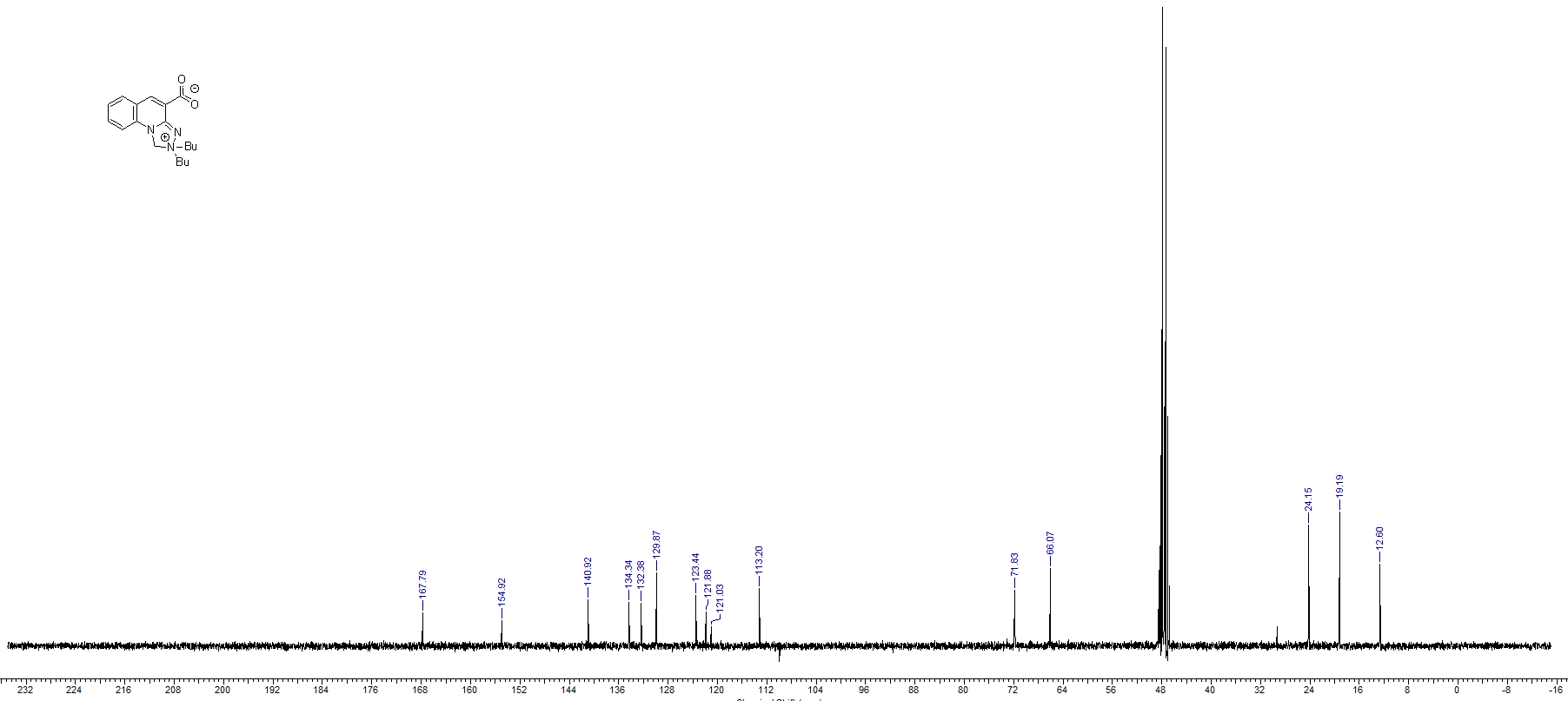


**Figure S4.** ^13^C-NMR (75 MHz, CD_3_OD) spectrum of 2,2-dibutyl-1,2-dihydro-[1,2,4]triazolo[4,3-*a*]quinolin-2-ium-4-carboxylate (**5c**).


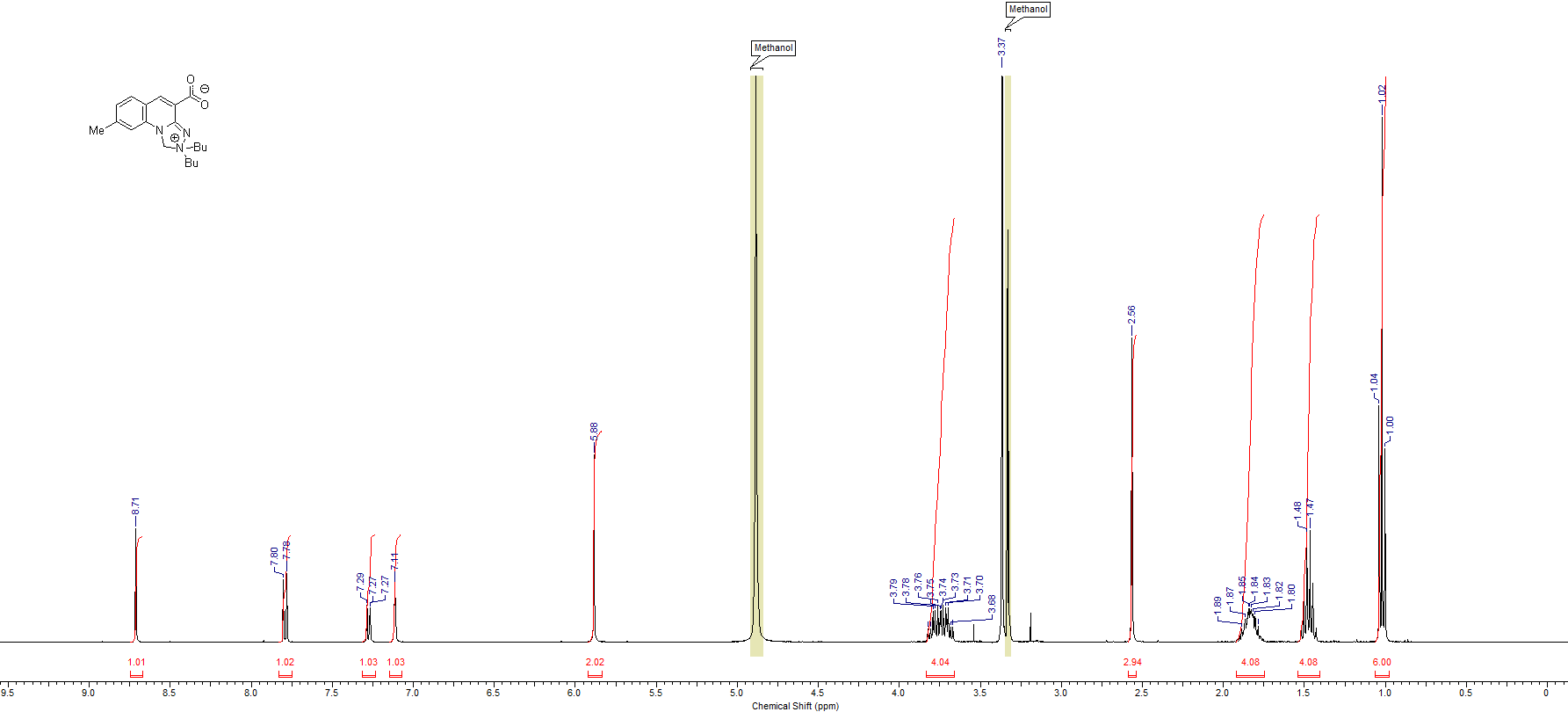


**Figure S5.** ^1^H-NMR (400 MHz, CD_3_OD) spectrum of 2,2-dibutyl-8-methyl-1,2-dihydro-[1,2,4]triazolo[4,3-*a*]quinolin-2-ium-4-carboxylate (**5q**).


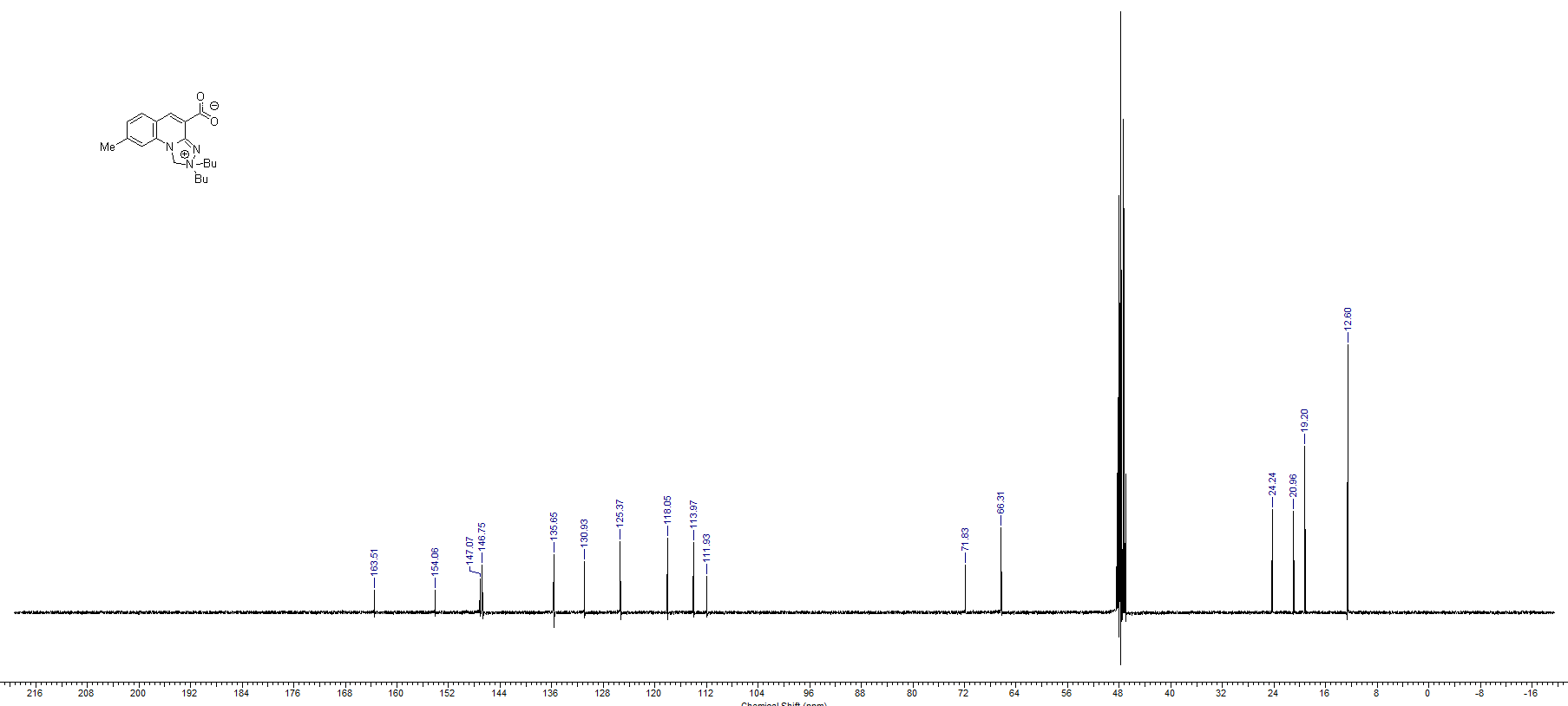


**Figure S6.** ^13^C-NMR (100 MHz, CD_3_OD) spectrum of 2,2-dibutyl-8-methyl-1,2-dihydro-[1,2,4]triazolo[4,3-*a*]quinolin-2-ium-4-carboxylate (**5q**)


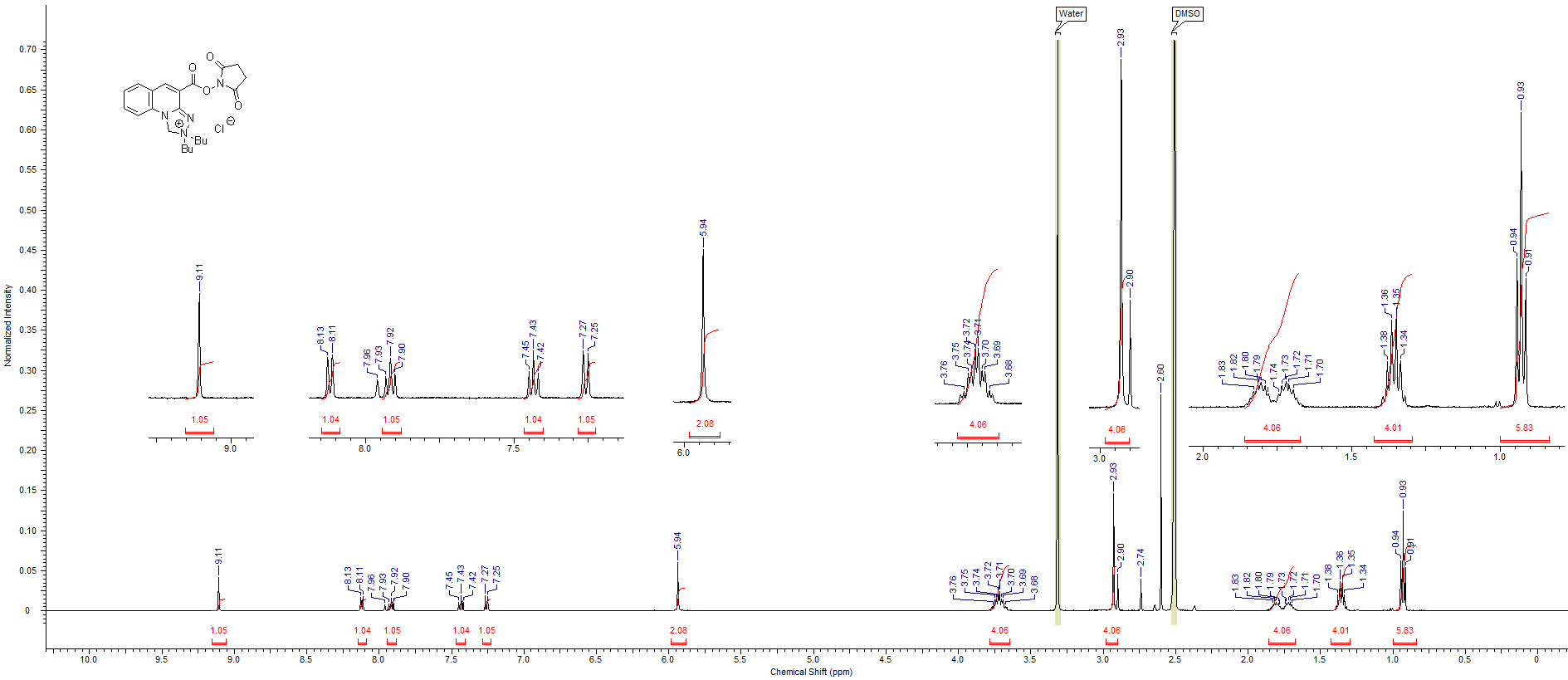


**Figure S7.** ^1^H-NMR (500 MHz, DMSO-*d_6_*) spectrum of 2,2-dibutyl-4-((2,5-dioxopyrrolidin-1-yloxy)carbonyl)-1,2-dihydro-[1,2,4]triazolo[4,3-*a*]quinolin-2-ium chloride (**6c**)


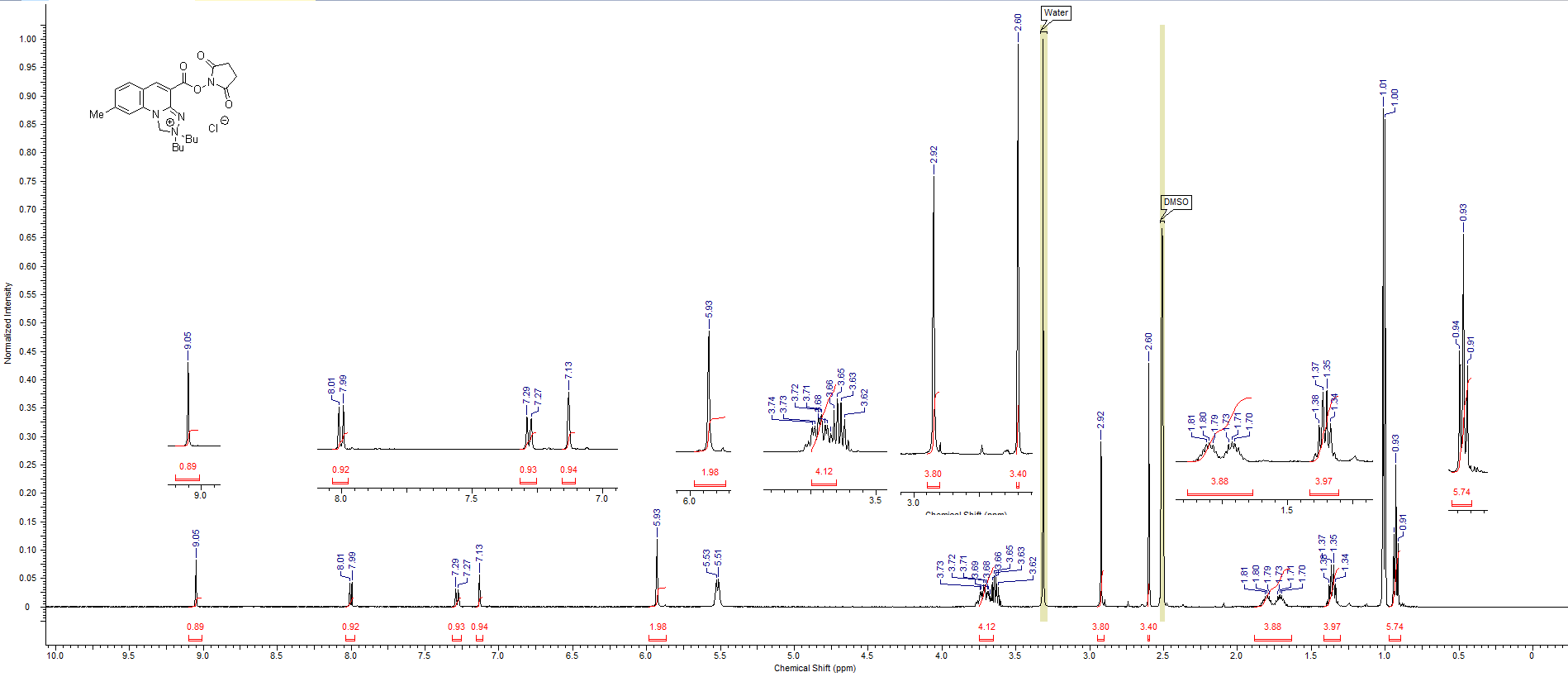


**Figure S8.** ^1^H-NMR (500 MHz, DMSO-*d_6_*) spectrum of 2,2-dibutyl-4-((2,5-dioxopyrrolidin-1-yloxy)carbonyl)-8-methyl-1,2-dihydro-[1,2,4]triazolo[4,3-*a*]quinolin-2-ium chloride (**6q**)


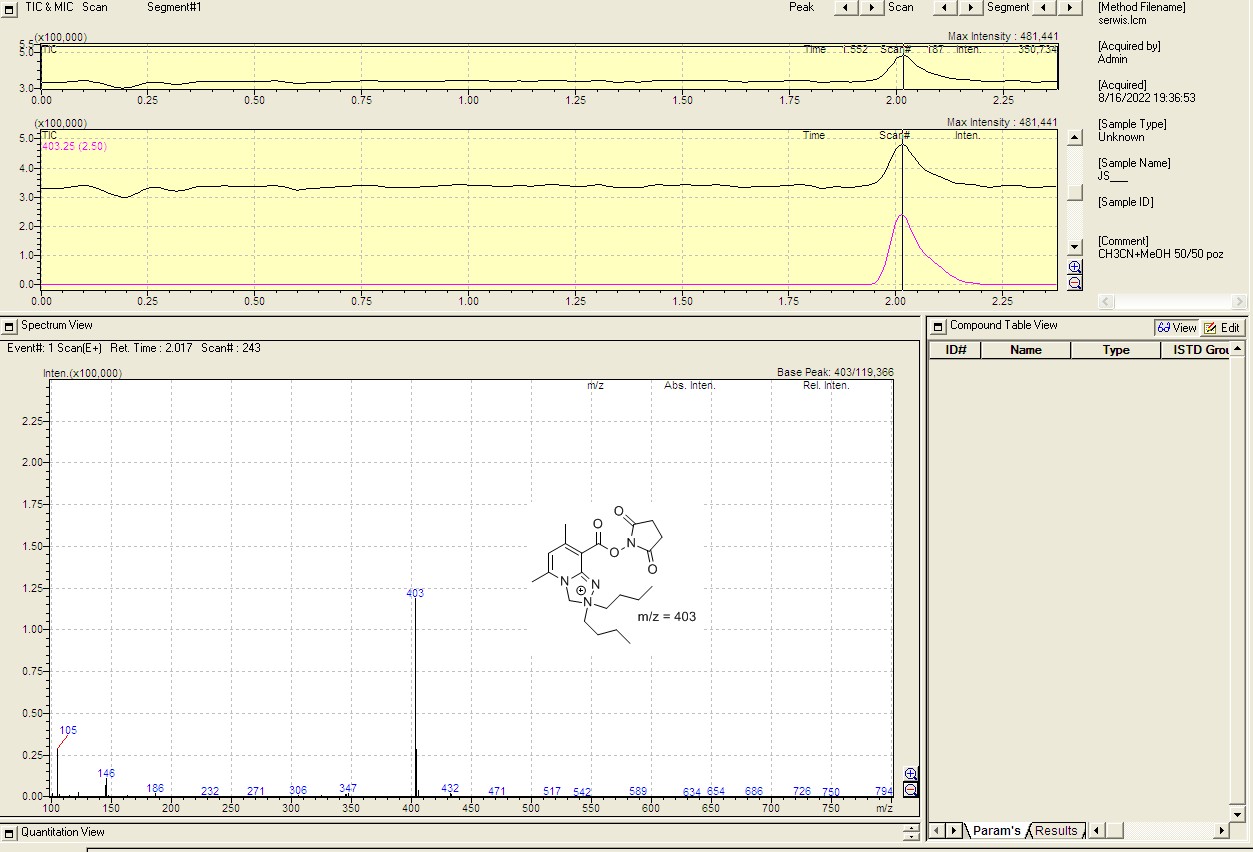


**Figure S9.** Mass spectrum of 2,2-Dibutyl-8-((2,5-dioxopyrrolidin-1-yloxy)carbonyl)-5,7-dimethyl-2,3-dihydro-[1,2,4]triazolo[4,3-a]pyridin-2-ium chloride (**3d**)


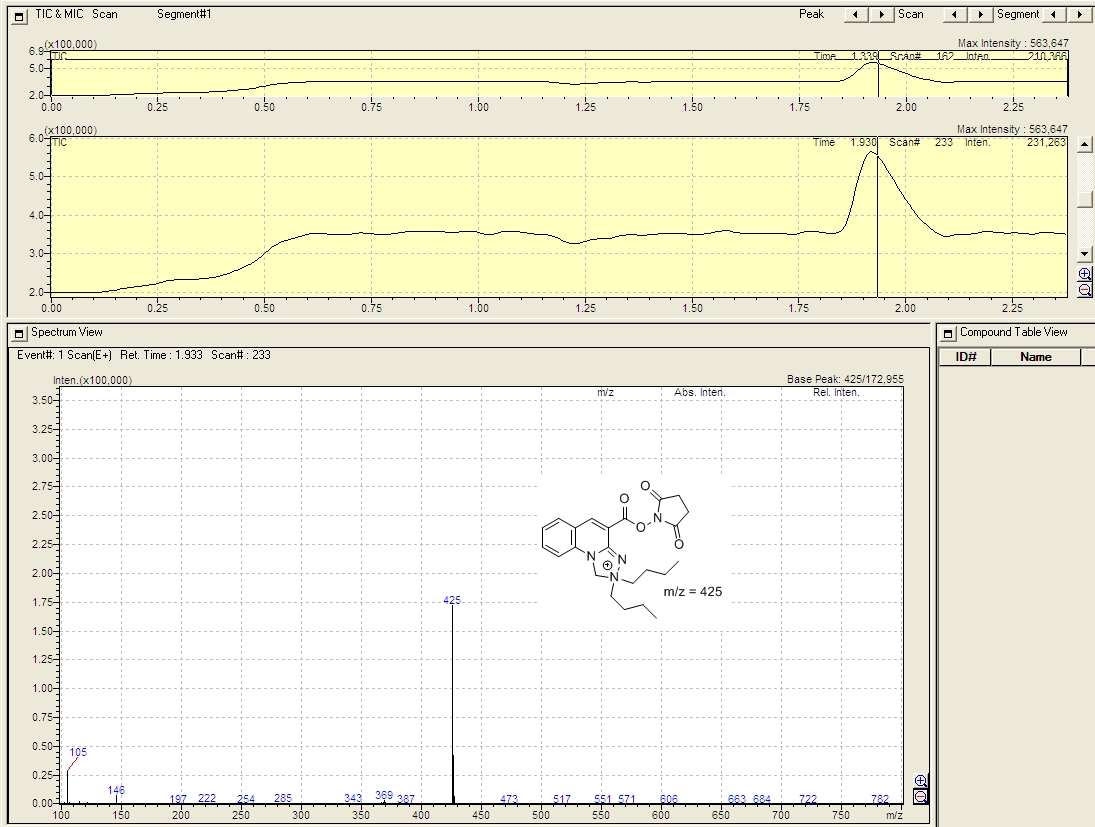


**Figure S10.** Mass spectrum of 2,2-dibutyl-4-((2,5-dioxopyrrolidin-1-yloxy)carbonyl)-1,2-dihydro-[1,2,4]triazolo[4,3-*a*]quinolin-2-ium chloride (**6c**)


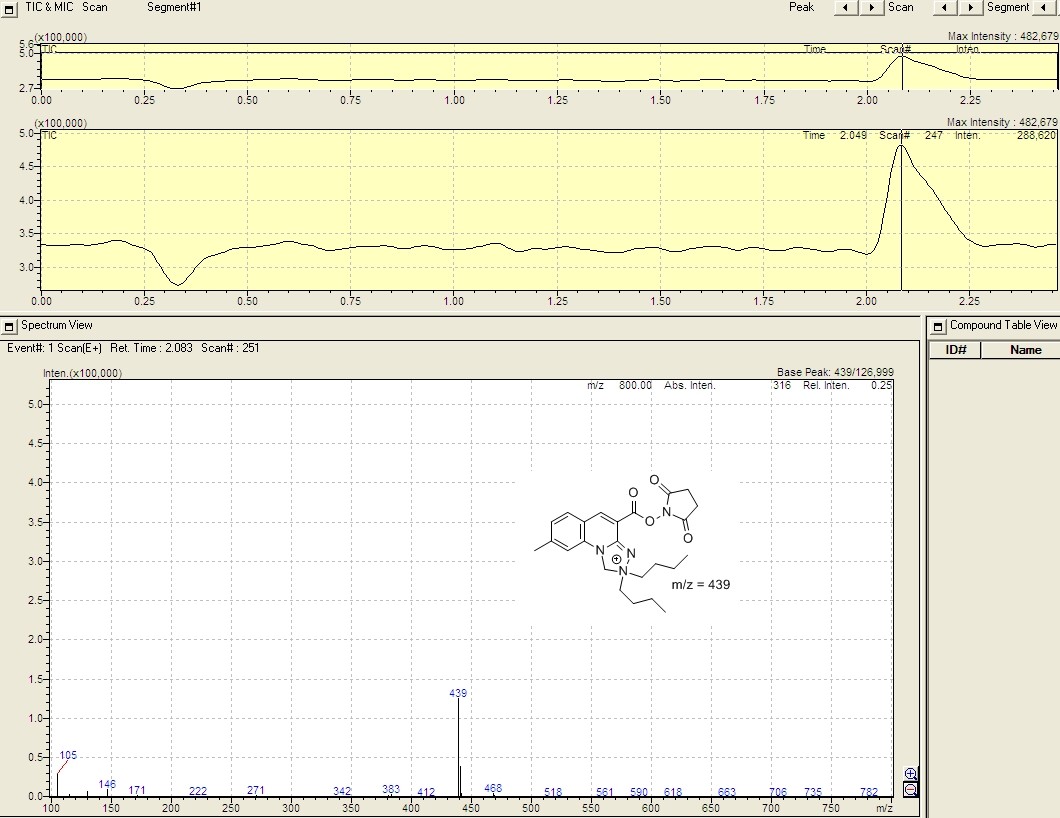


**Figure S11.** Mass spectrum of 2,2-dibutyl-4-((2,5-dioxopyrrolidin-1-yloxy)carbonyl)-8-methyl-1,2-dihydro-[1,2,4]triazolo[4,3-*a*]quinolin-2-ium chloride (**6q**)


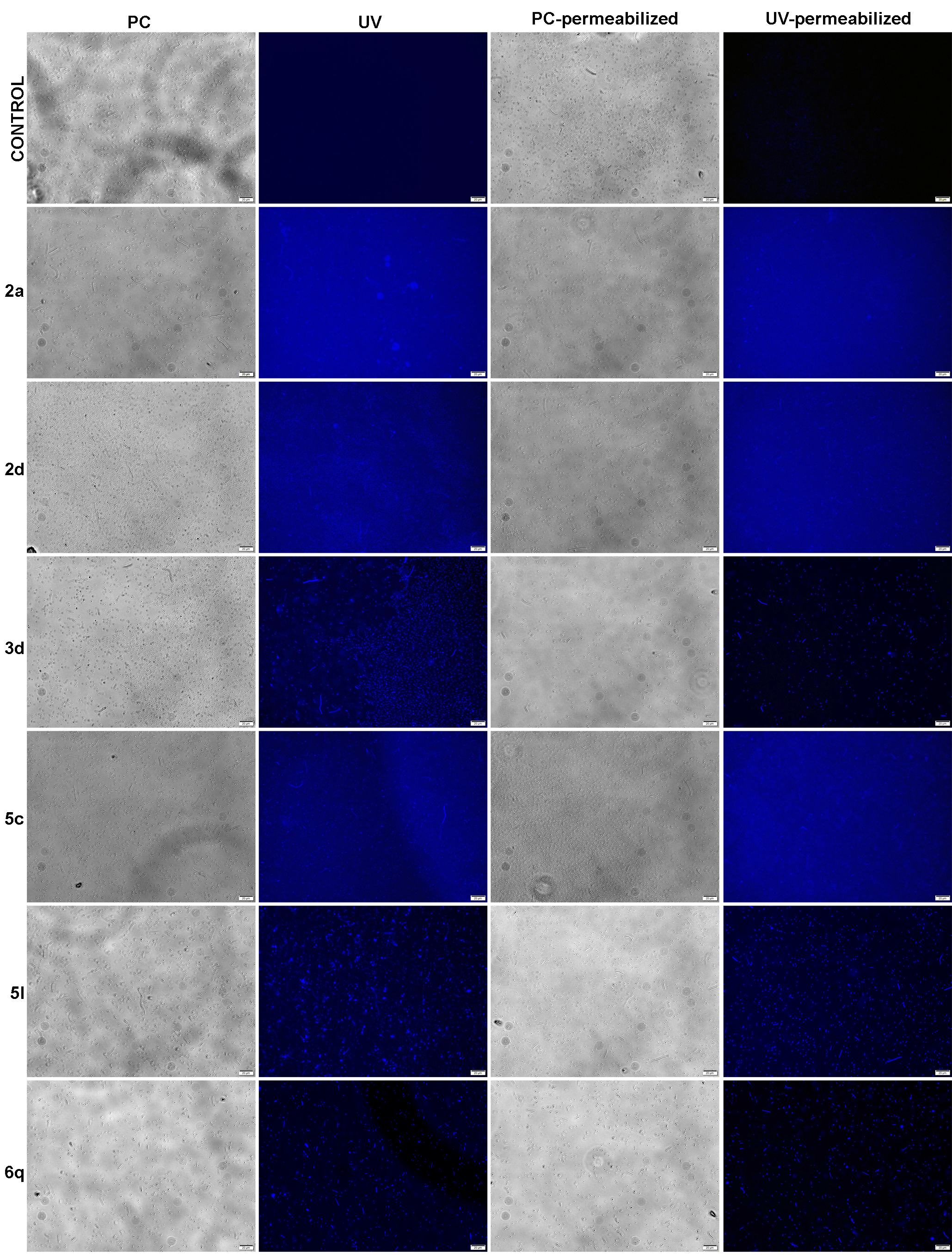


**Figure S12.** Visualization of free floating non-permeabilized and permeabilized *E. coli* BL21DE3 using phase-contrast microscopy and fluorescence microscopy with filter sets dedicated to DAPI (blue fluorescence) denoted as PC, UV, PC-permeabilized and UV-permeabilized, respectively. CONTROL – not stained *E. coli* BL21DE3. **2a**, **2d**, **3d**, **5c**, **5l**, and **6q** – *E. coli* BL21DE3 stained with the appropriate dye. Scale bars correspond to 20 µm.


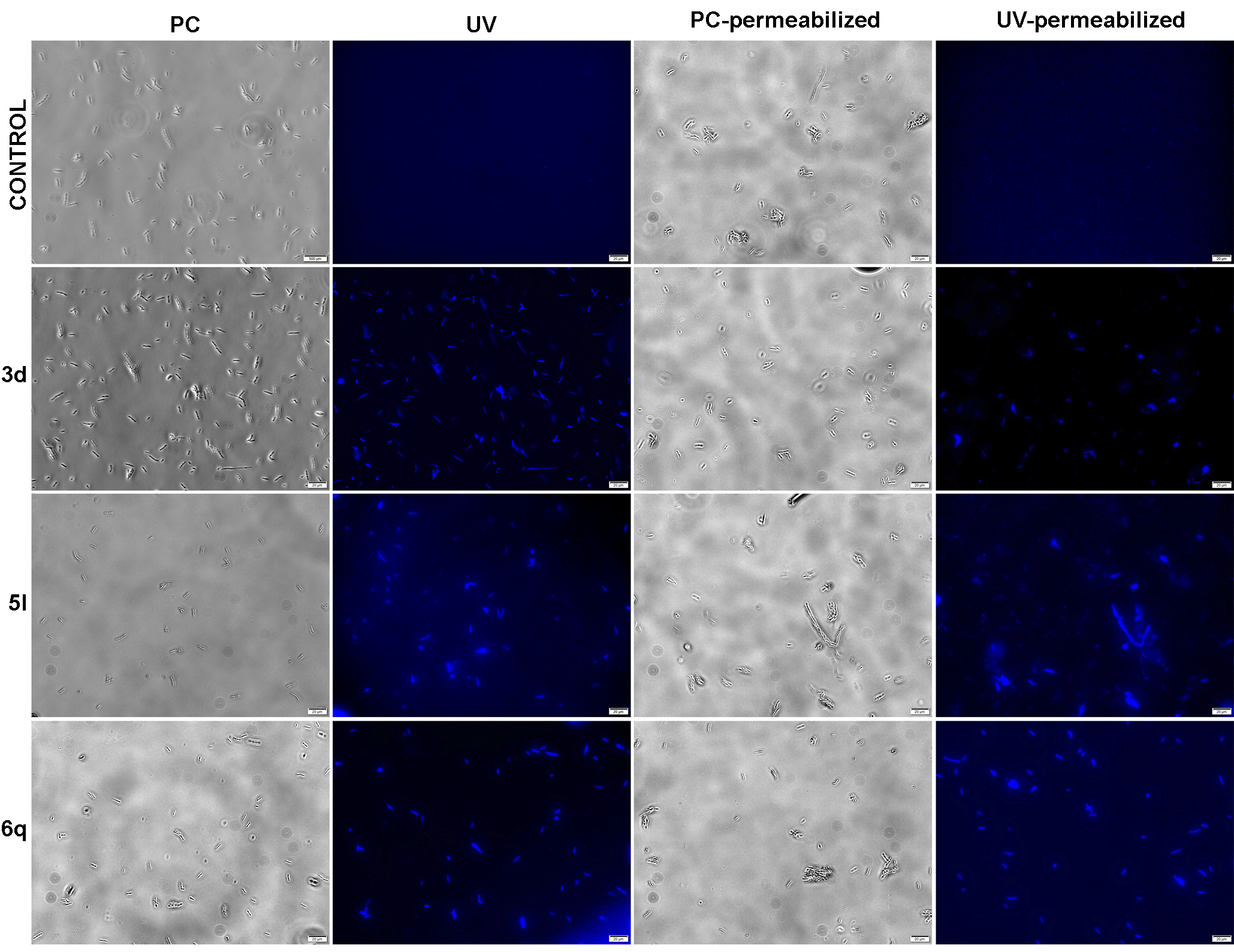


**Figure S13.** Visualization of free floating non-permeabilized and permeabilized *B. mycoides* using phase-contrast microscopy and fluorescence microscopy with filter sets dedicated to DAPI (blue fluorescence) denoted as PC, UV, PC-permeabilized and UV-permeabilized, respectively. CONTROL – not stained *B. mycoides*. **3d**, **5l** and **6q** – *B. mycoides* stained with the appropriate dye. Scale bars correspond to 20 µm.


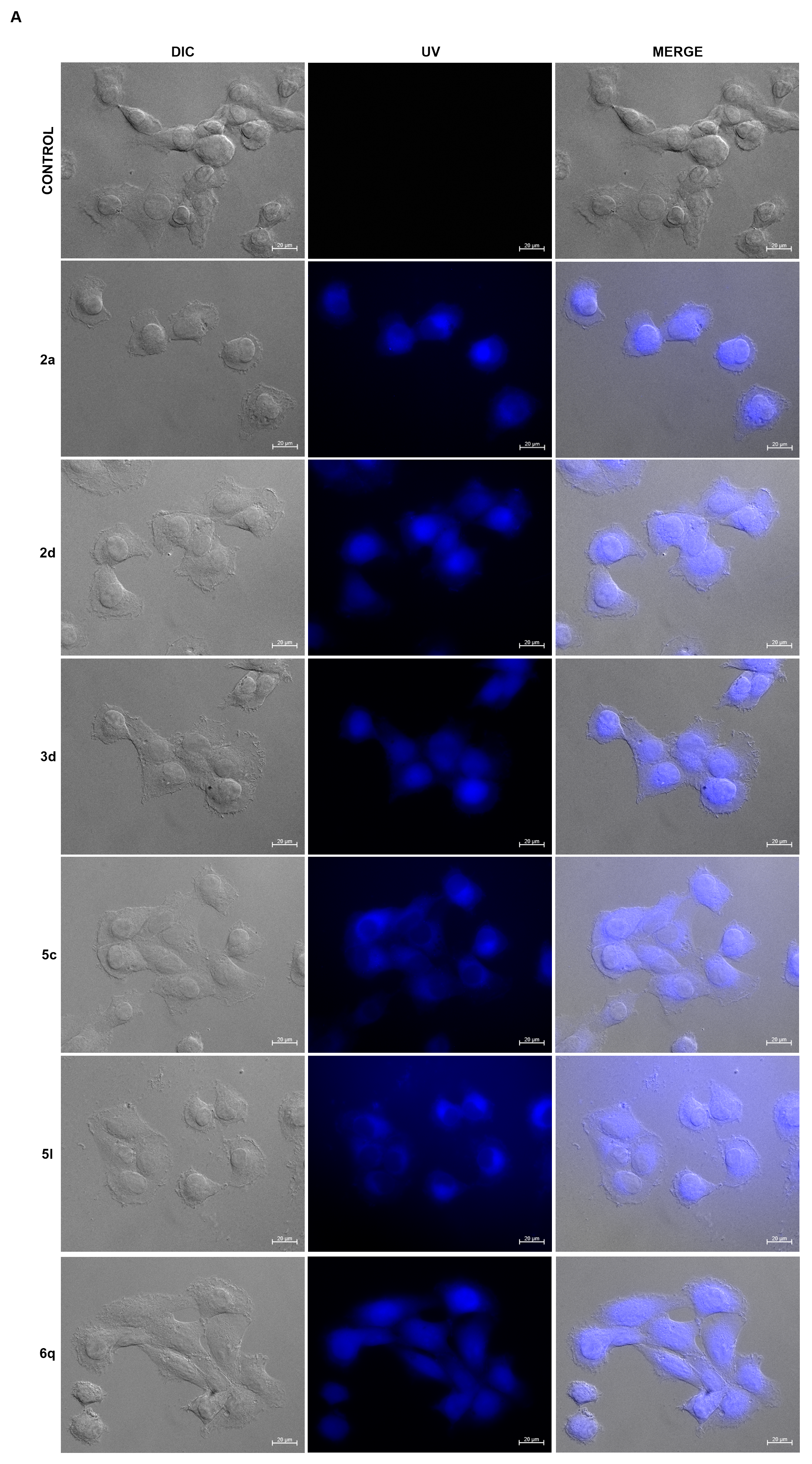


**Figure S14.** Differential interference contrast and fluorescence microscopy visualization of non-permeabilized HEK293 cells. CONTROL – cells incubated without dyes: **2a** (Ex: 115 s), **2d** (Ex: 64 s), **3d** (Ex: 0.105 s), **5c** (Ex: 90 s), **5l** (3 s) and **6q** (0.202 s) – cells stained with the appropriate. DIC – differential interference contrast microscopy, UV – fluorescence microscopy and Ex – Exposure Time. Scale bars correspond to 20 µm.


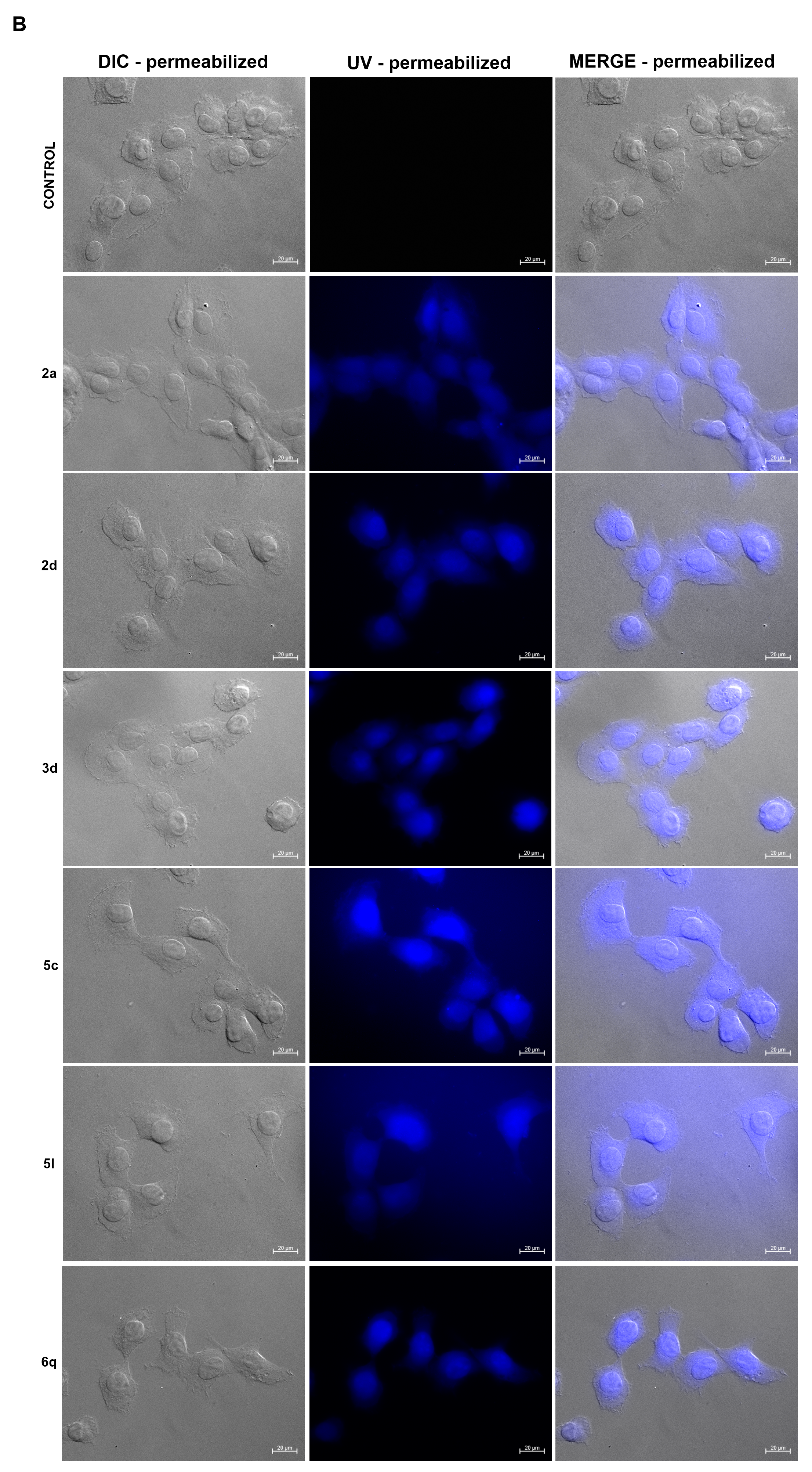


**Figure S15.** Differential interference contrast and fluorescence microscopy visualization of permeabilized HEK293 cells. CONTROL – cells incubated without dyes: **2a** (Ex: 100 s), **2d** (Ex: 61 s), **3d** (0.107 s), 5c (100 s), **5l** (6 s) and **6q** (0.323 s) – cells stained with the appropriate. DIC – differential interference contrast microscopy, UV – fluorescence microscopy and Ex – Exposure Time. Scale bars correspond to 20 µm.

**Table S1.** Retention factors for the investigated dyes obtained with RP and IAM HPLC

|  | **RP-HPLC** | | | | | | | **IAM-HPLC** | | | |
| --- | --- | --- | --- | --- | --- | --- | --- | --- | --- | --- | --- |
| **Compound** | Short gradient (0 to 15 minutes) | | Long gradient (0 to 30 minutes) | | mean time of short gradient | mean time of long gradient | log*k*_w_ | t*R*_1_ | t*R_2_* | meant*R* | CHI_IAM_ |
|  | t*R*_1_ | t*R_2_* | t*R*_1_ | t*R_2_* |  |  |  |  |  |  |  |
| **2a** | 1.91 | 1.87 | 1.98 | 1.98 | 1.89 | 1.98 | 1.11 | ND | ND | ND | ND |
| **2b** | 2.04 | 2.04 | 2.05 | 2.05 | 2.04 | 2.05 | 1.13 | ND | ND | ND | ND |
| **2c** | 2.29 | 2.29 | 2.73 | 2.73 | 2.29 | 2.73 | 1.22 | ND | ND | ND | ND |
| **2d** | 4.65 | 4.63 | 5.30 | 5.27 | 4.64 | 5.29 | 1.57 | 1.71 | 1.72 | 1.72 | 2.50 |
| **2e** | 13.91 | 13.89 | 23.76 | 23.73 | 13.90 | 23.74 | 5.37 | 4.09 | 4.09 | 4.09 | 39.20 |
| **2f** | 2.08 | 2.07 | 2.10 | 2.11 | 2.08 | 2.11 | 1.14 | ND | ND | ND | ND |
| **2g** | 2.33 | 2.38 | 2.42 | 2.42 | 2.36 | 2.42 | 1.19 | ND | ND | ND | ND |
| **2h** | 1.55 | 1.53 | 1.54 | 1.53 | 1.54 | 1.54 | 1.03 | ND | ND | ND | ND |
| **3b** | 2.07 | 2.04 | 2.07 | 2.08 | 2.06 | 2.08 | 1.13 | 2.96 | 2.98 | 2.98 | 11.60 |
| **3d** | 4.70 | 4.74 | 5.47 | 5.45 | 4.72 | 5.46 | 1.71 | 3.95 | 3.96 | 3.96 | 37.10 |
| **3e** | 14.55 | 14.44 | 26.78 | 26.76 | 14.50 | 26.77 | 5.92 | 5.78 | 5.78 | 5.78 | 65.10 |
| **5a** | 2.31 | 2.31 | 2.34 | 2.33 | 2.31 | 2.33 | 1.18 | ND | ND | ND | ND |
| **5b** | 3.15 | 3.13 | 3.28 | 3.28 | 3.14 | 3.28 | 1.58 | 1.72 | 1.72 | 1.72 | 2.70 |
| **5c** | 6.38 | 6.35 | 8.32 | 8.32 | 6.36 | 8.32 | 2.25 | 2.28 | 2.28 | 2.29 | 11.40 |
| **5d** | 13.36 | 13.43 | 22.75 | 22.82 | 13.40 | 22.79 | 5.36 | 4.32 | 4.32 | 4.32 | 42.70 |
| **5e** | 14.95 | 14.96 | 27.44 | 27.44 | 14.96 | 27.44 | 6.05 | 4.98 | 4.98 | 4.98 | 52.80 |
| **5f** | 4.66 | 4.55 | 5.34 | 5.34 | 4.60 | 5.34 | 1.81 | ND | ND | ND | ND |
| **5g** | 4.36 | 4.36 | 5.02 | 5.01 | 4.36 | 5.01 | 1.87 | 1.84 | 1.85 | 1.85 | 4.70 |
| **5h** | 2.93 | 2.97 | 3.03 | 3.02 | 2.95 | 3.03 | 1.27 | 1.72 | 1.71 | 1.71 | 2.50 |
| **5i** | 3.32 | 3.31 | 3.50 | 3.52 | 3.31 | 3.51 | 1.66 | 1.77 | 1.77 | 1.78 | 3.60 |
| **5j** | 2.25 | 2.25 | 2.36 | 2.36 | 2.25 | 2.36 | 1.18 | 2.37 | 2.37 | 2.37 | 12.70 |
| **5k** | 3.74 | 3.75 | 3.98 | 3.98 | 3.74 | 3.98 | 1.19 | 1.76 | 1.77 | 1.77 | 3.40 |
| **5l** | 4.69 | 4.59 | 5.33 | 5.33 | 4.64 | 5.33 | 1.67 | 1.97 | 1.97 | 1.97 | 6.60 |
| **5m** | 3.30 | 3.31 | 3.48 | 3.48 | 3.30 | 3.48 | 1.48 | 1.69 | 1.69 | 1.69 | 2.20 |
| **5n** | 3.89 | 3.87 | 4.29 | 4.31 | 3.88 | 4.30 | 1.77 | 1.83 | 1.83 | 1.83 | 4.50 |
| **5o** | 8.63 | 8.62 | 12.55 | 12.56 | 8.62 | 12.56 | 2.77 | 2.91 | 2.92 | 2.92 | 21.10 |
| **5p** | 4.57 | 4.58 | 5.31 | 5.30 | 4.58 | 5.30 | 1.82 | 1.98 | 1.97 | 1.97 | 6.60 |
| **5q** | 7.65 | 7.66 | 10.77 | 10.88 | 7.65 | 10.82 | 2.52 | 2.63 | 2.63 | 2.64 | 16.80 |
| **5r** | 6.14 | 6.12 | 7.85 | 7.87 | 6.13 | 7.86 | 2.13 | 2.32 | 2.30 | 2.31 | 11.70 |
| **6b** | 3.49 | 3.48 | 3.76 | 3.76 | 3.48 | 3.76 | 1.81 | 1.73 | 1.73 | 1.73 | 2.45 |
| **6c** | 6.52 | 6.47 | 8.56 | 8.55 | 6.50 | 8.56 | 2.28 | 2.29 | 2.30 | 2.30 | 11.60 |
| **6d** | 13.95 | 13.94 | 23.86 | 23.86 | 13.94 | 23.86 | 5.49 | 4.33 | 4.32 | 4.33 | 42.70 |
| **6p** | 4.72 | 4.65 | 5.46 | 5.44 | 4.69 | 5.45 | 1.77 | 1.98 | 1.99 | 1.99 | 6.80 |
| **6q** | 8.09 | 7.91 | 11.41 | 11.33 | 8.00 | 11.37 | 2.64 | 3.63 | 3.63 | 3.63 | 32.10 |
|  |  |  |  |  |  |  |  |  |  |  |  |
| Fluorescein | 6.94 | 6.92 | 8.33 | 8.33 | 6.93 | 8.33 | 2.55 | 3.09 | 3.09 | 3.09 | 23.80 |
| Sulforhodamine B | 3.07 | 3.13 | 4.97 | 5.03 | 3.10 | 5.00 | 1.52 | 3.35 | 3.35 | 3.35 | 27.80 |

**Table S2.** Wavelengths of maximum absorbance and emission determined in 0.1M H_2_SO_4_ water solution. The color of the circle corresponds to the color of fluorescence at the wavelength of maximum emission.

| **Compounds** | **Wavelengths of maximum absorbance [nm]** | **Wavelengths of maximum emission [nm]** |
| --- | --- | --- |
| **2a-h**, **3b**,**d**,**e** | 343±2 | 449±1 **O** |
| **5a-5k**, **6b-d** | 373±3 | 462±2 **O** |
| **5l** | 379 | 469 **O** |
| **5m** | 375 | 474 **O** |
| **5n** | 394 | 491 **O** |
| **5o** | 387 | 485 **O** |
| **5p**,**q**, **6p**,**q** | 373±1 | 457±1 **O** |
| **5r** | 378 | 466 **O** |

The photophysical properties of compounds **2b, 5b**,**l-r** were reported previously [Saczewski, J.; Fedorowicz, J.; Sączewski, J.; Drażba, Z.; Wiśniewska, P.; Gdaniec, M.; Wicher, B.; Suwiński, G.; Jalińska, A. Synthesis and fluorescence of dihydro-[1,2,4]triazolo[4,3-a]pyridin-2-iumcarboxylates: An experimental and TD-DFT comparative study. *Dyes Pigment*. **2019**, *161*, 347–359. (10.1016/j.dyepig.2018.09.005)].
